# Nontoxic *N*-Heterocyclic Olefin Catalyst Systems for Well-Defined Polymerization of Biocompatible Aliphatic Polycarbonates

**DOI:** 10.1021/acspolymersau.2c00017

**Published:** 2022-07-25

**Authors:** Christian Czysch, Thi Dinh, Yannick Fröder, Leon Bixenmann, Patric Komforth, Alexander Balint, Hans-Joachim Räder, Stefan Naumann, Lutz Nuhn

**Affiliations:** † Max Planck Institute for Polymer Research, Ackermannweg 10, 55128 Mainz, Germany; ‡ Institute of Polymer Chemistry, University of Stuttgart, Pfaffenwaldring 55, 70569 Stuttgart, Germany; § Chair of Macromolecular Chemistry, Julius-Maximilians-Universität Würzburg, Röntgenring 11, 97070 Würzburg, Germany

**Keywords:** Aliphatic polycarbonate, biocompatible, cocatalysis, N-heterocyclic olefins, organocatalysis, polycarbonate, polymer micelle, postpolymerization modification, ring-opening polymerization, transesterification

## Abstract

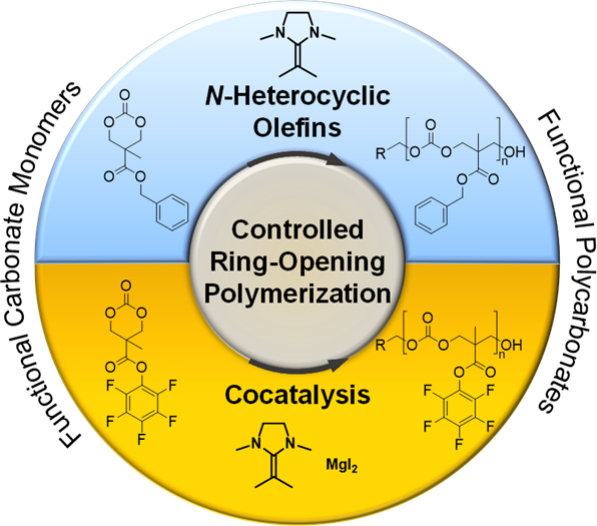

Herein, *N*-heterocyclic olefins (NHOs) are utilized as catalysts for the ring-opening polymerization (ROP) of functional aliphatic carbonates. This emerging class of catalysts provides high reactivity and rapid conversion. Aiming for the polymerization of monomers with high side chain functionality, six-membered carbonates derived from 2,2-bis(hydroxymethyl)propionic acid (bis-MPA) served as model compounds. Tuning the reactivity of NHO from predominant side chain transesterification at room temperature toward ring-opening at lowered temperatures (−40 °C) enables controlled ROP. These refined conditions give narrowly distributed polymers of the hydrophobic carbonate 5-methyl-5-benzyloxycarbonyl-1,3-dioxan-2-one (MTC-OBn) (*Đ* < 1.30) at (pseudo)first-order kinetic polymerization progression. End group definition of these polymers demonstrated by mass spectrometry underlines the absence of side reactions. For the active ester monomer 5-methyl-5-pentafluorophenyloxycarbonyl-1,3-dioxane-2-one (MTC-PFP) with elevated side chain reactivity, a cocatalysis system consisting of NHO and the Lewis acid magnesium iodide is required to retune the reactivity from side chains toward controlled ROP. Excellent definition of the products (*Đ* < 1.30) and mass spectrometry data demonstrate the feasibility of this cocatalyst approach, since MTC-PFP has thus far only been polymerized successfully using acidic catalysts with moderate control. The broad feasibility of our findings was further demonstrated by the synthesis of block copolymers for bioapplications and their successful nanoparticular assembly. High tolerability of NHO in vitro with concentrations ranging up to 400 μM (equivalent to 0.056 mg/mL) further emphasize the suitability as a catalyst for the synthesis of bioapplicable materials. The polycarbonate block copolymer mPEG_44_-*b*-poly(MTC-OBn) enables physical entrapment of hydrophobic dyes in sub-20 nm micelles, whereas the active ester block copolymer mPEG_44_-*b*-poly(MTC-PFP) is postfunctionalizable by covalent dye attachment. Both block copolymers thereby serve as platforms for physical or covalent modification of nanocarriers for drug delivery.

## Introduction

The necessity to use metal-free catalysts in bioapplications has led to the development of a variety of new organocatalysts for ring-opening polymerization (ROP).^1−3^ These novel catalyst systems extend the properties of conventional metal catalysts such as high reaction rates and control with respect to improved biocompatibility.^4^ Thereby, organocatalysts are able to bridge the gap of providing defined synthesis and a low toxicity profile, allowing the application of various polymeric materials in the biological context.^5^ In this emerging class of catalysts enzymes, Brønsted acids and bases as well as Lewis acids and bases were implemented, giving a plethora of tools to polymer chemists.^2,3^ Especially the Brønsted base 1,8-diazabicyclo[5.4.0]undec-7-ene (DBU) and *N*-heterocyclic carbenes (NHCs) as a class of Lewis bases were further explored for the ring-opening of lactones and cyclic carbonates due to their high activity and specificity.^6−9^

The class of *N*-heterocyclic olefins (NHOs) recently emerged as a powerful alternative, and several types of polymers were synthesized in a rapid, highly controlled manner.^10−13^ Most notably, ring-opening of epoxides was achieved, yielding high molecular weights of up to >10^6^ g·mol^–1^ and with rapid turnover.^14^ In addition, the successful synthesis of polylactones (l-lactide, ε-caprolactone, and δ-valerolactone) and polycarbonates (trimethylene carbonate, TMC) was accomplished by fine-tuning the NHO reactivity based on their structure, basicity, and steric hindrance.^15^ By using cocatalyst systems^16^ of NHOs and Lewis acids, e.g., metal halides, the reactivity is even further adjustable to the specific needs.^10,17−19^ To date, NHO polymerization is limited to nonfunctional materials, as the high reactivity of NHOs complicates the introduction of additional functions and results in undesirable side reactions. Therefore, controlled polymerization of functionalized monomers has not been achievable so far.

Especially for biological applications, functional polymers are needed to manufacture tailor-made materials.^20,21^ Thus, tuning polymer functionalization provides the ability (a) to anchor or encapsulate therapeutic molecules,^22,23^ (b) to enable a (triggered/stimuli-responsive) release,^24,25^ and (c) to fine-tune polymer degradation.^26,27^ Aliphatic polycarbonates are a promising material class in this regard due to their functionalizability by side chain introduction, biodegradability, and favorable toxicological profiles.^6,21,28,29^ Polycarbonate-based block copolymers were self-assembled into nanoparticular carriers and used for a variety of therapeutic applications.^30−33^ An important motif for functional polycarbonates are six-membered monomers derived from 2,2-bis(hydroxymethyl)propionic acid (bis-MPA) with a carboxy side chain substituent which, through esterification, can comprise a wide range of different functional groups.^6,34^ An important monomer within this group is the hydrophobic 5-methyl-5-benzyloxycarbonyl-1,3-dioxan-2-one (MTC-OBn) that fosters block copolymer self-assembly into micellar nanoparticles and the encapsulation of hydrophobic (aromatic) compounds (supported by π–π stacking).^33,35^ Alternatively, the development and polymerization of 5-methyl-5-pentafluorophenyloxycarbonyl-1,3-dioxane-2-one (MTC-PFP), a six-membered carbonate monomer with an active ester side chain motif, by Hedrick and co-workers enlarged the polycarbonate toolbox.^36,37^ However, previously ROP of MTC-PFP was only achieved by using an acidic catalyst (e.g., trifluoromethanesulfonic acid) which, however, provided polymers of only moderate definition.^37^ Starting from poly(MTC-PFP) postpolymerization, modification reactions access various materials by amine conjugation.^38,39^ Polymeric materials accessed from both monomers were shown to be highly tolerable in vitro^33^ and even in vivo,^40^ and they can therefore be considered as biocompatible.

By selecting the functional carbonate monomers MTC-OBn and MTC-PFP for our study, we aimed to investigate the influence of ester side chain motifs on the polymerization process under NHO catalysis. Since organobase ROP catalysts, such as NHOs, often also display pronounced transesterification activity, ester side chains are not fully orthogonal functionalities and, therefore, pose a major challenge for the controlled ROP of carbonates.^11,41^ Due to its high side chain reactivity, the active ester monomer MTC-PFP was chosen as an appealing, yet challenging monomer to study the NHO-guided catalyst system. Overall, we intended to extend NHO-based catalysis to the controlled chain growth polymerization of functional monomers and to further demonstrate the feasibility of this approach with respect to accessing micellar nanocarriers, which allow encapsulation or covalent conjugation of molecules for drug delivery.

## Results and Discussion

**Figure 1:**
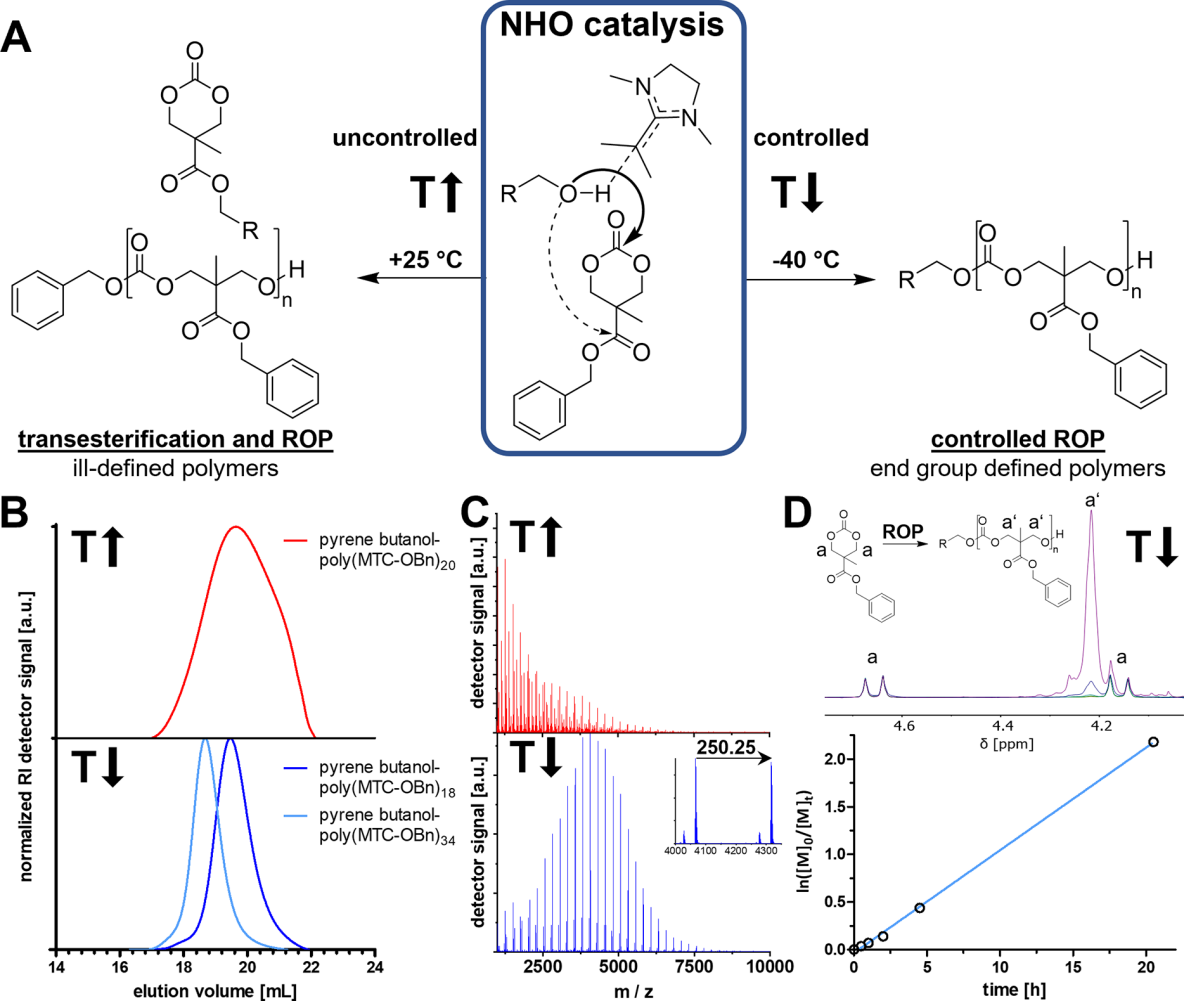
*N*-Heterocyclic olefins (NHO) as ring-opening polymerization (ROP) catalysts for functional six-membered carbonate 5-methyl-5-benzyloxycarbonyl-1,3-dioxan-2-one (MTC-OBn). (A) Temperature-dependent tuning of catalyst activity from ROP and simultaneous transesterification at room temperature (RT, +25 °C) to selective ROP without side reactions at decreased temperatures (−40 °C). (B) SEC traces of homopolymers initiated by pyrene butanol showing broad distribution at RT (red trace) and narrow distributions at −40 °C (blue traces), recorded via refractive index (RI). (C) Mass spectrometric analysis of pyrene butanol-poly(MTC-OBn)_20_. Reactions at RT (red) provide various side products and a broad distribution compared to ROP at −40 °C with defined end groups (blue). Species deriving from two different ionization mechanisms were identified (photoionization with smaller intensities and K^+^ cationization with larger intensities) with steps of 250.25 *m*/*z* (corresponding to the monomer mass of MTC-OBn). (D) Kinetic evaluation of controlled ROP of MTC-OBn at −40 °C targeting a DP of 40. Monitoring the conversion by ^1^H NMR spectroscopy (top graph) with steady growth of polymer signal (a’) and simultaneous decline of monomeric signal (a) reaching conversions of 88.7%. Kinetic plot of conversion (bottom graph) as analyzed by ^1^H NMR spectroscopy with linear reaction kinetics of pseudo-first-order, thereby providing controlled reaction propagation (*y* = 0.1055·h^–1^·*x*, *R*^2^ = 0.998).

For the establishment of NHO-catalyzed ROP of functional six-membered carbonates, the NHO 1,3-dimethyl-2-(1-methylethylidene)imidazolidine was employed, which was previously identified as a controlled catalyst in the ROP of the archetypal carbonate TMC.^15^ First experiments focused on the polymerization of MTC-OBn and aimed at gaining a deeper insight into general reaction parameters such as concentration and temperature (Figure 1A). Reactions at nonoptimized conditions (room temperature, 0.2 M in THF) demonstrated the rapid conversion by NHO catalysts but yielded ill-defined polymers. The resulting products were analyzed by size exclusion chromatography (SEC) showing moderate distributions (*Đ* = 1.80, Figure 1B, top), and detailed analysis by MALDI-ToF spectrometry revealed the formation of diverse molecular architectures as products (Figure 1C, top). In addition to the desired polymer (pyrene butanol-poly(MTC-OBn)) further products are formed by side reactions (see Figure S1 for detailed mass analysis and Figure S2 for NMR spectra). Transesterification at the side chain leads to the release of benzyl alcohol, resulting in polymer chains with benzyl alcohol as initiating end group (benzyl alcohol-poly(MTC-OBn)) which limits achievable high molecular weights and control over the end groups. Further side reactions such as backbiting by transesterification of hydroxy chain ends at the ester groups of the polymer side chain and at the polycarbonate backbone could be identified as well. Backbiting reactions yield ring-closed polymers that have lost their reactive hydroxy end group and therefore create nonpropagating chains, which further deteriorate the polymerization outcome. Yet, the nonoptimized polymerization attempt still confirmed the ability of the NHO to catalyze ring-opening of functional six-membered carbonates, however, the observed side reactions limit control and demand further improvements.

**Table 1:** Summary of NHO-Catalyzed ROP of MTC-OBn at 0.2 M in THF Initiated by Pyrene Butanol

	*T* [°C]	χ_n_^targ^	conv [%]^a^	*M*_n_^theo^ [g/mol]^a^	*M*_n_^prod^ [g/mol]^a^	*M*_n_^prod^ [g/mol]^b^	*Đ*^b^
pyrene butanol-poly(MTC-OBn)_20_	+25	20	87.5	4650	5280	3120	1.80
pyrene butanol-poly(MTC-OBn)_18_	–40	20	83.5	4450	4750	4240	1.29
pyrene butanol-poly(MTC-OBn)_34_	–40	40	88.7	9150	8860	8430	1.21

a Determined by ^1^H NMR analysis.

b Determined by hexafluoroisopropanol (HFIP) size exclusion chromatography, calibrated with PMMA standards.

Fortunately, superior polymerization conditions were found when lowering the reaction temperature. Polymerizations at −40 °C favored ROP to transesterification, thereby allowing the rapid synthesis of defined pyrene butanol-poly(MTC-OBn) (Figure S3 for NMR spectra). The strong influence of temperature became evident by the drastically narrowed dispersity (*Đ*) from 1.80 (at +25 °C) to 1.29 (at −40 °C) (Figure 1B, bottom and Figure S4). Even more clearly, MALDI-ToF spectrometry shows poly(MTC-OBn) with the desired pyrene-butanol initiator group and hydroxy end groups, proving the end-group defined polymerization of MTC-OBn using NHO (Figure 1C, bottom and Figure S5) and the absence of side reactions. By adjusting the reaction parameters, we tuned the catalytic behavior of NHOs and redirected its high activity from transesterification at the side chain toward the ring-opening of the cyclic six-membered carbonate (see Table 1 for summary).

To further demonstrate the controlled manner of NHO-catalyzed ROP of MTC-OBn, we investigated the kinetic profile of the reaction by taking samples from the reaction solution at defined intervals and analyzing them (Figure 1D). Continuous chain growth was evidenced by increasing conversion (determined by ^1^H NMR spectroscopy) during reaction time, and high conversions of ∼90% were found after 20 h (Figure 1D, top). Even more significantly, ROP of MTC-OBn proceeds with (pseudo)first-order kinetics as shown by the kinetic plot of the conversion data (Figure 1D, bottom and Figure S6).

**Figure 2:**
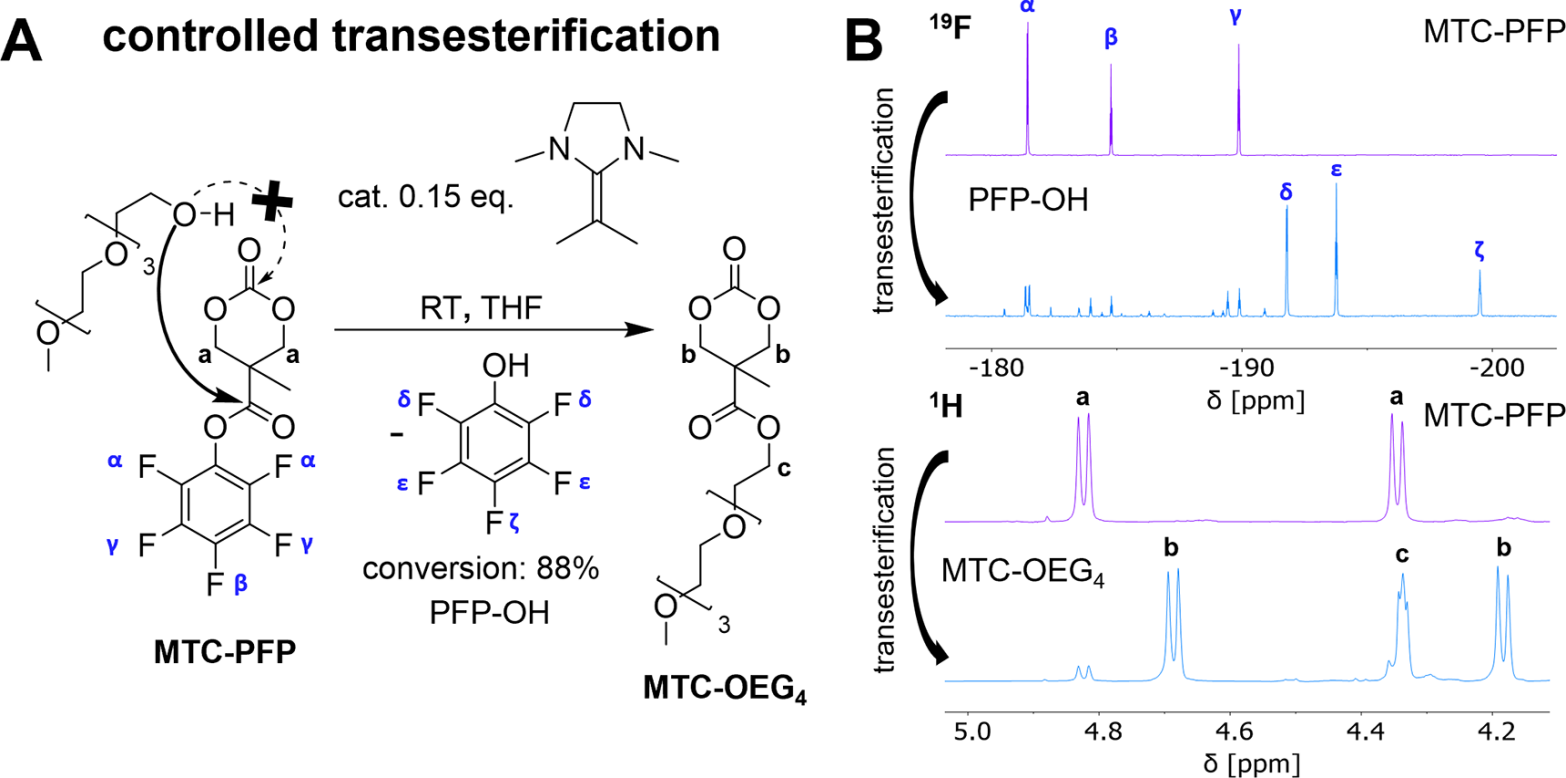
NHO-catalyzed transesterification of active ester carbonate monomer 5-methyl-5-pentafluorophenyloxycarbonyl-1,3-dioxane-2-one (MTC-PFP) by side chain reaction with alcohols. (A) Under the conditions of the controlled ROP for MTC-OBn, a polymerization does not occur. However, selective transesterification is observed and exploited for the synthesis of ester functional monomers (tetraethylene glycol monomethyl ether). (B) ^19^F NMR spectra (top) showing high conversion of 88% by declining PFP ester signals (α, β, γ) and rising signals of released PFP–OH (δ, ε, ζ). ^1^H NMR spectra (bottom) underlining the formation of the tetraethylene glycol ester product by a shift of the methylene signals from a (educt) to b (product) and the occurrence of alpha methylene protons by the formed ester c.

Encouraged by these excellent results, we attempted the polymerization of the reactive ester monomer MTC-PFP (Figure 2). However, when analogous reaction conditions were applied, no polymerization was observed by NMR spectroscopy (Table 2). Analysis of the recorded ^19^F NMR spectra revealed a minor conversion of the active ester side chains, indicated by released pentafluorophenol (PFP), when monomer, catalyst, and initiator were mixed. Due to the higher side chain reactivity of MTC-PFP compared to MTC-OBn, the same reaction conditions in this case did not result in polymerization but in consumption of initiating alcohols through transesterification reactions. Subsequently, the non-nucleophilic PFP is released which does not initiate polymer chain growth. Although the initial goal of MTC-PFP polymerization could not be achieved, the observed high regiospecificity of the side chain reaction attracted our attention, since it could enable a straightforward synthesis of bis-MPA-based monomers with functional side group substituents.

Therefore, we investigated the feasibility of NHO-catalyzed transesterification for the reaction with tetraethylene glycol monomethyl ether (Figure 2A). High conversions of ∼88%, as investigated by ^19^F NMR spectroscopy, underline the catalytic potential and regiospecificity of NHOs for transesterification of MTC-PFP (Figure 2B). Respective signals of the formed 5-methyl-5-tetraethylene glycol monomethyl ether carbonyl-1,3-dioxan-2-one (MTC-OEG_4_) product were then identified by ^1^H NMR spectroscopy (Figure 2B, bottom and Figure S7) and demonstrated the feasibility of this approach for the straightforward generation of MTC monomers.

**Figure 3:**
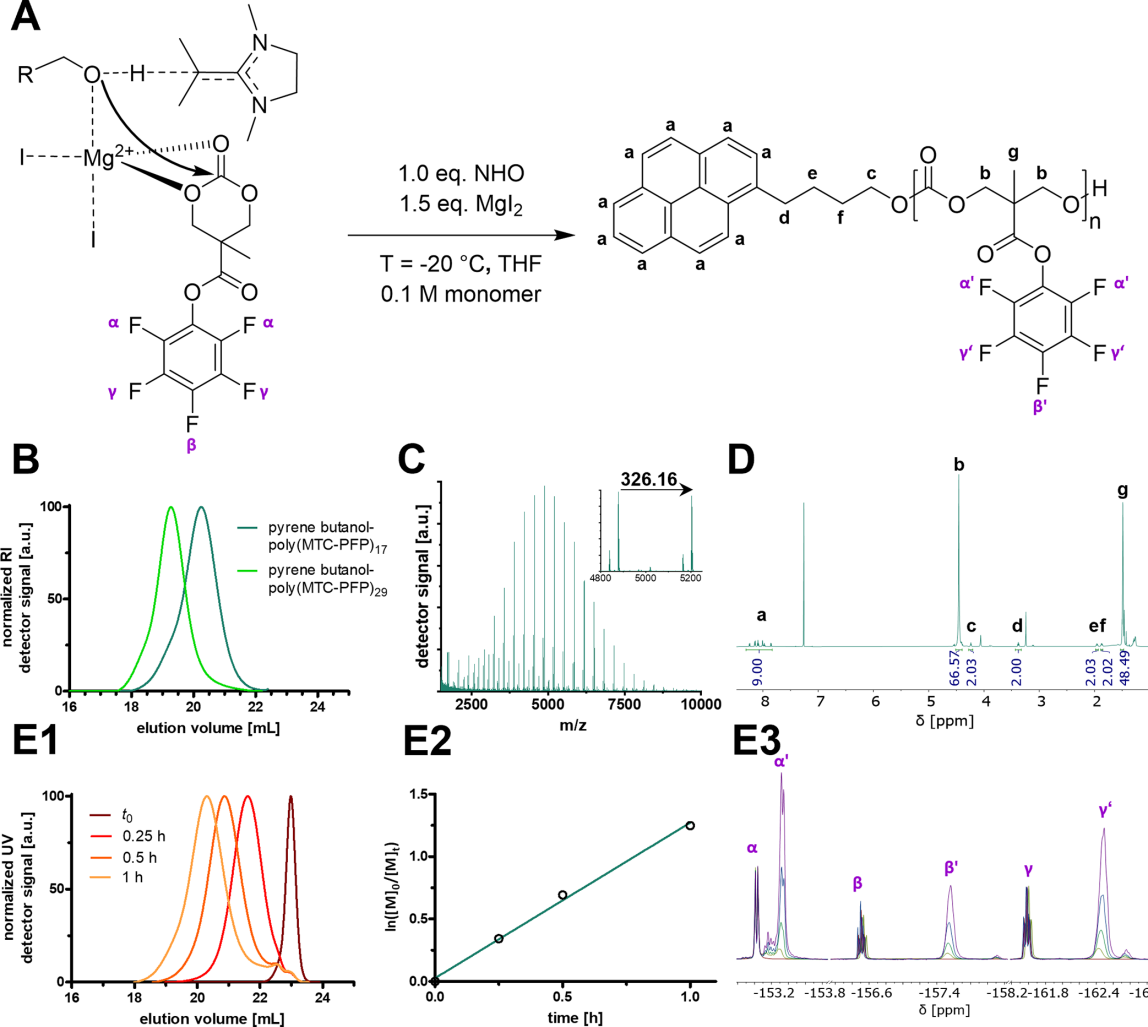
Controlled ROP of reactive ester monomer MTC-PFP by cocatalysis using NHO and Lewis acid magnesium iodide. (A) Proposed catalysis mechanism: By cooperative action by NHO and coordination by MgI_2_, propagating chain ends are directed toward ROP instead of transesterification. (B) SEC traces of homopolymers initiated at −20 °C by pyrene butanol with narrow distributions recorded by refractive index (RI). (C) Mass spectrometric analysis of pyrene butanol-poly(MTC-PFP)_17_ showing the high definition of cocatalyzed ROP at −20 °C yielding polymers with defined end groups (green). Species deriving from two different ionization mechanisms were identified (photoionization, smaller intensity, and K^+^ cationization, larger intensity) with steps of 326.16 *m*/*z* (corresponding to the monomer mass of MTC-PFP). (D) ^1^H NMR spectrum of pyrene butanol-poly(MTC-PFP)_17_ homopolymer. (E) Kinetic evaluation of controlled ROP of MTC-PFP at −20 °C targeting a DP of 20. (E1) SEC analysis (UV detector) of MTC-PFP polymerization at various time points. (E2) Kinetic plot of conversion analyzed by ^19^F NMR spectroscopy providing linear reaction kinetics of pseudo-first-order, thereby providing controlled reaction propagation (*y* = 1.2805·h^–1^·*x*, *R*^2^ = 0.998). (E3) Monitoring the conversion by ^19^F NMR spectroscopy with steady growth of polymer signals (α’, β’, γ’) and simultaneous decline of monomeric signals (α, β, γ) reaching monomer conversion of ∼80% after 2 h.

Previous investigations highlighted that the cocatalysis of NHOs with Lewis acids holds a great potential for the modulation of ROP.^10,17−19,42,43^ Having established that a direct polymerization of MTC-PFP using solely NHO is impossible, we therefore attempted a cocatalyst approach employing the Lewis acid magnesium iodide (MgI_2_). This specific metal halide has been found to significantly increase polymerization control and to moderate NHO reactivity for lactone conversion.^43^ We thus opted for this particular Lewis acid to prevent transesterification but retune NHO activity toward ROP. Similar to the ROP of MTC-OBn, the cocatalytic approach for MTC-PFP was attempted at decreased temperature and low monomer molarity (Figure 3A). This time, the polymerization of the six-membered carbonate was evident by NMR spectroscopy, indicating that cocatalysis indeed shapes the reactivity of NHOs from transesterification toward ROP. Narrow dispersities (*Đ* ≈ 1.25) of the obtained polymers highlight the controlled manner of the cocatalyzed polymerization (Figure 3B; note that for SEC the catalyst had to be removed to avoid signal overlap, as demonstrated by Figure S8). Detailed mass analysis of the polymers by MALDI-ToF spectrometry confirmed the end group integrity (Figures 3C, S9, and 1S0). Successful preparation of homopolymers with targeted DP of 20 and 40 showed the high control over molar mass and molecular integrity (Figures 3B–D and S11). We have for the first time conclusively shown the ROP of MTC-PFP with a nonacidic catalyst system (see Table 2 for summary). The novel cocatalyst system serves as an additional tool in the ROP catalyst toolbox and extends applications of MTC-PFP to e.g., combinations with acid-labile polymeric architectures.

Finally, a kinetic evaluation was performed by taking samples from the NHO/MgI_2_-cocatalyzed MTC-PFP polymerization at several time points (Figure 3E). Polymer chain growth was followed by a time-dependent increase in molecular weight, as determined by SEC (Figure 3E1), and a steady increase of monomer conversion, as determined by NMR spectroscopy (Figure 3E3 for ^19^F and Figure S12 for ^1^H NMR spectra). Analysis of the obtained data revealed again a (pseudo)-first-order kinetic (Figure 3E2).

In summary, we have shown that NHO itself does not allow ROP of MTC-PFP but rather catalyzes transesterification at the side chain, which enables the synthesis of new monomers. Also, the Lewis acid magnesium iodide alone does not trigger any ROP at the chosen reaction conditions (Figures S13 and S14). In contrast, when both are applied as a NHO-metal halide cocatalyst system, the reactivity is directed toward the cyclic carbonate functionality, as proposed by the reaction mechanism of Figure 3A. We hypothesize that the interaction of the Lewis acid with MTC-PFP’s more electron-rich carbonate group is presumably favored over the interaction with the less electron-dense active ester carbonyl which may also be sterically less accessible. Simultaneously, the Lewis acid can further coordinate the alcohol for its nucleophilic attack toward ROP. The cocatalyst itself therefore seems to regiospecifically switch the reactivity. Altogether, using two different approaches for the polymerization of functional six-membered carbonates, in case of MTC-OBn by tuning the reaction temperature and in case of MTC-PFP by further applying a cocatalyst system, NHOs can be applied for controlled ROP of functional monomers.

Considering an application of the obtained materials in a biomedical context, we aimed to explore the suitability of NHOs and therefore tested the biocompatibility of the catalyst. Residual amounts of the catalyst might potentially remain in the material and would then be applied along with the material.^5^ In that respect, we checked for the cell viability of NHO by treating a macrophage cell line with NHO concentrations of up to 400 μM (equivalent to 0.056 mg/mL). Fortunately, no adverse effects on cell metabolism could be found by MTT (3-(4,5-dimethylthiazol-2-yl)-2,5-diphenyltetrazolium bromide) assay after 24 h (Figure S15). In addition, the cocatalyst’s magnesium salts have already been applied in several medicinal contexts^44^ and its abundance in biological environments^45^ suggests a high tolerability of the Lewis acid magnesium iodide.

**Table 2:** Summary of NHO/MgI_2_ Cocatalyzed ROP of MTC-PFP at 0.1 M in THF Initiated by Pyrene Butanol

	*T* (°C)	χ_n_^targ^	conv [%]^a^	*M*_n_^theo^ [g/mol]^a^	*M*_n_^prod^ [g/mol]^a^	*M*_n_^prod^ [g/mol]^b^	*Đ*^b^
pyrene butanol-poly(MTC-PFP)	–20 (without MgI_2_)	20	0	–	–	–	–
pyrene butanol-poly(MTC-PFP)	–20 (without NHO)	20	0	–	–	–	–
pyrene butanol-poly(MTC-PFP)_17_	–20	20	81.4	5580	5700	2760	1.24
pyrene butanol-poly(MTC-PFP)_29_	–20	40	69.7	9100	9740	5460	1.25

a Determined by ^1^H NMR analysis.

b Determined by hexafluoroisopropanol (HFIP) size exclusion chromatography, calibrated with PMMA standards.

**Figure 4:**
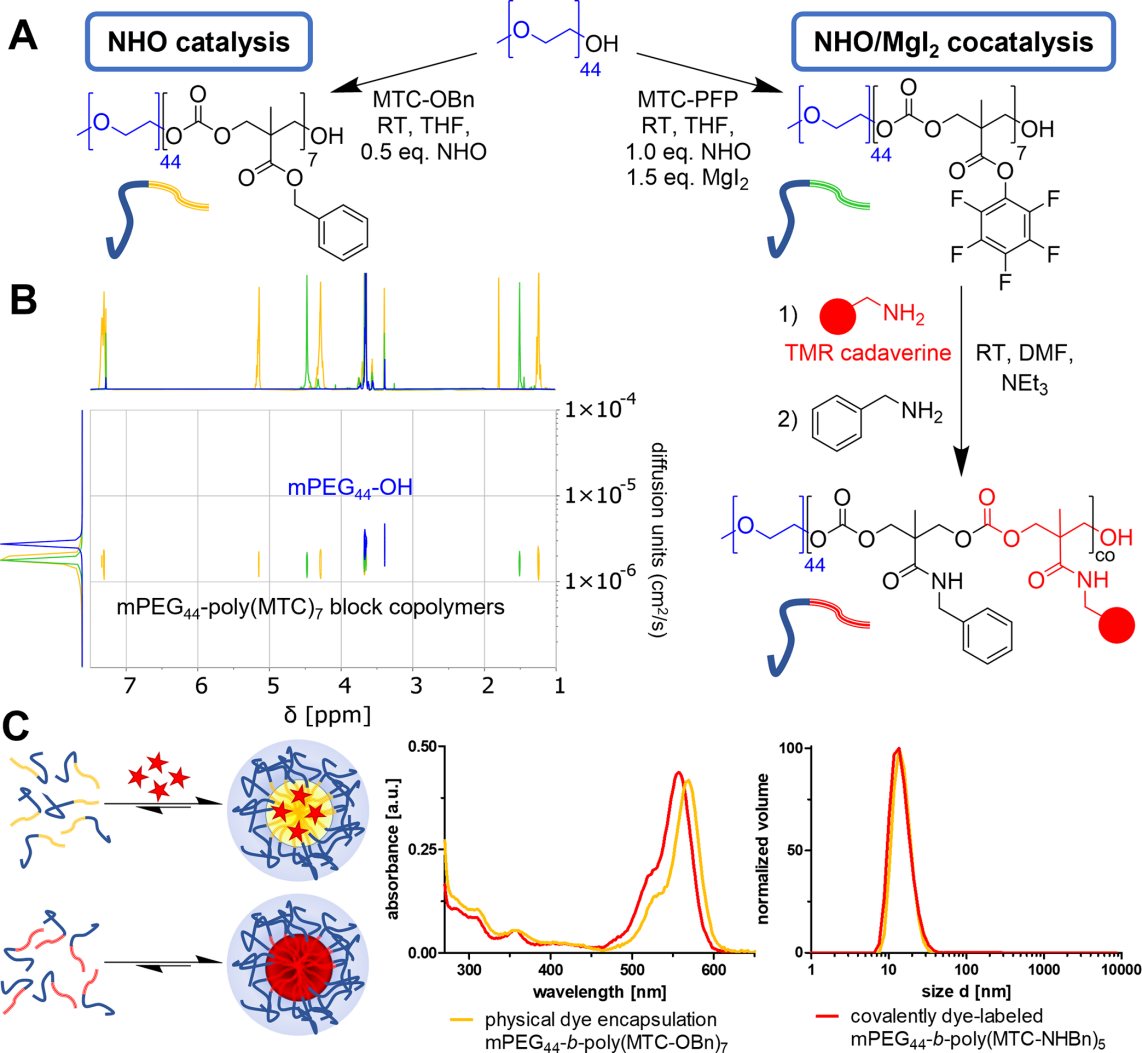
Block copolymer synthesis for the fabrication of dye-loded and dye-labeled polymeric micelles. (A) NHO-catalyzed synthesis of mPEG_44_-*b*-poly(MTC-OBn) (left) and NHO/MgI_2_ cocatalyzed synthesis of mPEG_44_-*b*-poly(MTC-PFP) (right), followed by postpolymerization modification with TMR cadaverine and benzyl amine to access mPEG_44_-*b*-poly(MTC-NHBn). (B) ^1^H DOSY NMR spectra of mPEG_44_–OH macro initiator (blue) block copolymers mPEG_44_-*b*-poly(MTC-OBn)_7_ (yellow) and mPEG_44_-*b*-poly(MTC-PFP)_7_ (green) confirming the growth of the polycarbonate block from mPEG_44_ by respective signals. Moreover, lower diffusion coefficients of the block copolymer indicate increased molecular weights. (C) Micellar self-assembly of block copolymer. For mPEG_44_-*b*-poly(MTC-OBn)_7_ the dye octadecyl rhodamine B dye can simultaneously be encapsulated (left, top sketch) by solvent-evaporation method. Analogously, covalently dye-labeled mPEG_44_-*b*-poly(MTC-NHBn)_5_ assembles into micelles as well (left, bottom sketch). UV–vis spectra (middle) of mPEG_44_-*b*-poly(MTC-OBn)_7_ micelles with physical dye encapsulation (yellow) and covalently dye-labeled mPEG_44_-*b*-poly(MTC-NHBn)_5_ micelles (red). DLS particle size distribution (right) shows similar micelles with a volume mean of 15.1 nm for mPEG_44_-*b*-poly(MTC-OBn)_7_ micelles (yellow, PDI = 0.03) and 15.3 nm for mPEG_44_-*b*-poly(MTC-NHBn)_5_ micelles (red, PDI = 0.48).

For accessing nanosized drug delivery systems, amphiphilic block copolymers are of high interest due to their ability to self-assemble into micellar nanoparticles. To demonstrate the general suitability of functional materials synthesized by NHO organocatalysis for these purposes, we next aimed to prepare amphiphilic polycarbonate-based block copolymers. Therefore, we adapted the previous polymerization parameters and initiated polymerization by polyethylene glycol (mPEG_44_-OH) to yield polyethylene glycol-polycarbonate block copolymers (Figure 4A). Using the highly hydrophobic MTC-OBn, we prepared amphiphilic mPEG_44_-*b*-poly(MTC-OBn) block copolymers (see Figures S16, S17, and S21 for further characterization data and Table 3). Grafting of the polycarbonate block from mPEG_44_-OH was further confirmed by ^1^H DOSY NMR spectroscopy (Figure 4B). MTC-PFP-based block copolymers (mPEG_44_-*b*-poly(MTC-PFP)) were synthesized using the NHO cocatalyst system and gave similar results as mentioned for MTC-OBn (see also Figures S18–S21 for further characterization data and Table 3). The resulting poly(MTC-PFP) block copolymer had a similar diffusion behavior by ^1^H DOSY NMR spectroscopy as the poly(MTC-OBn) block copolymer (Figure 4B, both showing higher diffusion coefficients compared to mPEG_44_). The unique property of the active ester polymer to undergo an additional postpolymerization modification was leveraged by covalent attachment of a water-soluble amine-functional fluorescent dye (tetramethylrhodamine cadaverine, TMR). Subsequently, all remaining active ester sites of the dye-labeled polymer were converted using benzyl amine to yield an amphiphilic mPEG_44_-*b*-poly(MTC-NHBn) block copolymer that was purified by dialysis to remove PFP and then freeze-dried to gain a red voluminous powder. The conversion of the PFP-ester could be monitored by ^19^F NMR during the aminolysis reaction, while the isolated poly(MTC-NHBn) block copolymer did not provide any fluorine signals anymore demonstrating quantitative modification of the MTC-PFP block (Figure S22). In principle, the active ester approach therefore allows the conjugation of further cargos to the polymer chains such as amine-functional therapeutic drug molecules and is, thus, highly versatile for the generation of tailor-made drug carrying nanoparticles.

For nanoparticle formation block copolymers of mPEG_44_-*b*-poly(MTC-OBn) and mPEG_44_-*b*-poly(MTC-NHBn) were, respectively, self-assembled to form polymeric micelles by the solvent evaporation method.^33^ Using this method, polymer chains are first dissolved in acetone and then added dropwise to water. Upon evaporation of the volatile acetone, polymeric micelles are formed due to the high water solubility of the mPEG_44_ block and the insolubility of the benzyl ester/amide polycarbonate block. Physical interactions of the highly hydrophobic polycarbonate block stabilize the micelles and can entrap hydrophobic cargos. Addition of the hydrophobic dye octadecyl rhodamine B to mPEG_44_-*b*-poly(MTC-OBn) in the initial acetone solution thereby provides access to dye containing micelles.

Both particle solutions were subsequently characterized by DLS analysis, and successful particle aggregation was confirmed by hydrodynamic diameters of ∼15 nm (by volume mean, Figure 4C, right; note that some high molecular weight products that were probably formed during polymerization at room temperature by transesterification did not affect the self-assembly into well-defined and narrowly distributed micelles, compare Figure S23). Further micelle characterization by UV–vis spectroscopy further proved either the covalent dye-labeling of mPEG_44_-*b*-poly(MTC-NHBn) or the hydrophobic dye encapsulation of mPEG_44_-*b*-poly(MTC-OBn), as for both particles an additional dye-absorption at long wavelengths was found by UV–vis spectroscopy (Figure 4C, middle). Altogether these additional results convincingly demonstrate that polycarbonate block copolymers yielded by NHO-(co)catalyzed ROP are suitable for the fabrication of functional polymeric micelles. Combined with the high biocompatibility of NHO in vitro (Figure S15), the overall concept provides access to functional polycarbonate-based materials with promising properties for future drug delivery applications.

**Table 3:** Summary of Block Copolymer Synthesis by NHO-Catalyzed ROP for mPEG_44_-*b*-poly(MTC-OBn) and by NHO/MgI_2_ Catalyzed ROP at 0.2 M (for MTC-OBn) or 0.1 M (for MTC-PFP) Monomer Concentration in THF Initiated by mPEG_44_-OH at Room Temperature

	χ_n_^targ^	conv [%]^a^	*M*_n_^theo^ [g/mol]^a^	*M*_n_^prod^ [g/mol]^a^	*M*_n_^prod^ [g/mol]^b^	*Đ*^b^
mPEG_44_-*b*-poly(MTC-OBn)_4_	5	88.7	3110	2930	19290	1.21
mPEG_44_-*b*-poly(MTC-OBn)_7_	10	83.8	4100	3820	19240	1.13
mPEG_44_-*b*-poly(MTC-PFP)_5_	5	75.7	3230	3630	19290	1.17
mPEG_44_-*b*-poly(MTC-PFP)_7_	10	63.7	4080	4380	19440	1.14

a Determined by ^1^H NMR analysis.

b Determined by hexafluoroisopropanol (HFIP) size exclusion chromatography, calibrated with PMMA standards.

## Conclusion

In conclusion, we herein reported on the NHO-catalyzed polymerization of functional monomers. After reaction optimization, this organocatalytic approach allows defined syntheses of ester functional polycarbonates derived from bis-MPA. For the benzyl ester derivative MTC-OBn, lowering polymerization temperature was the key to tune NHO’s catalytic activity from transesterification at the side chain substituent toward ROP at the cyclic carbonate motif. Thereby, we accomplished controlled polymerizations yielding end group defined polymers. However, application of these polymerization conditions to a monomer with an activated ester side group (MTC-PFP) resulted exclusively in transesterification. Leveraging the high regiospecificity, we then demonstrated the NHO-catalyzed transesterification of MTC-PFP as a route to generate ester functional monomers. With the aim of directing the reactivity from transesterification toward ring-opening polymerization, we implemented a cocatalyst system. By using NHO and the Lewis acid magnesium iodide, we achieved controlled ROP of MTC-PFP, which was previously only possible by acid catalysis. The cocatalyst approach not only provides higher control and end group definition but also perspectively allows for the combination of acid-labile monomers/initiators with the highly versatile MTC-PFP monomer. Moving further toward biological application, we confirmed the low toxicity of the NHO organocatalyst in vitro and were thus encouraged to use the corresponding polymers for the preparation of polymeric micelles. By synthesizing amphiphilic block copolymers grafting onto mPEG_44_, we afforded amphiphilic mPEG_44_-*b*-poly(MTC-OBn) block copolymers and self-assembled dye-containing micelles by the solvent evaporation method. Using the active ester monomer, mPEG_44_-*b*-poly(MTC-PFP) block copolymers were yielded by NHO-metal halide cocatalysis. Subsequently, these precursor polymers were covalently equipped with the amine-functional fluorescent dye tetramethyl rhodamine cadaverine and remaining active esters were converted with benzyl amine. Dye-labeled mPEG_44_-*b*-poly(MTC-NHBn) was then also assembled into polymeric micelles, complementing the fabrication approach of physical dye-loaded mPEG_44_-*b*-poly(MTC-OBn) with a covalent attachment approach. Overall, we thereby demonstrated, to the best of our knowledge, for the first time that NHO catalysis is a feasible way to polymerize functional monomers and to even generate reactive postfunctionalizable polymers. The nanoparticular assembly of these polymers opens a wide range of possibilities for biomedical applications founding on the rapid and controlled NHO-catalyzed ROP of functional cyclic carbonates.

## Supporting Information Available

Details on the materials and methods as well as syntheses protocols and further characterization data (PDF)
