# Supplementary material for: Nontoxic N-Heterocyclic Olefin Catalyst Systems for Well-Defined Polymerization of Biocompatible Aliphatic Polycarbonates
Source: ACS Polym Au. 2022 Jul 25;2(5):371–9. doi: 10.1021/acspolymersau.2c00017 (PMC9955374; doi:10.1021/acspolymersau.2c00017)
Supplement: Supplementary file 1 — lg2c00017_si_001.pdf [file lg2c00017_si_001.pdf]

Non-toxic *N*-heterocyclic olefin catalyst systems for well-defined polymerization of biocompatible aliphatic polycarbonates

– Supporting Information –

*Christian Czysch,[1] Thi Dinh,[1] Yannick Fröder,[1] Leon Bixenmann,[1] Patric Komforth,[1] Alexander Balint,[2] Hans-Joachim Räder,[1] Stefan Naumann,[2] Lutz Nuhn[1,3,]**

1: Max Planck Institute for Polymer Research, Ackermannweg 10, 55128 Mainz, Germany
2: Institute of Polymer Chemistry, University of Stuttgart, Pfaffenwaldring 55, 70569 Stuttgart, Germany.
3: Chair of Macromolecular Chemistry, Julius Maximilian University Würzburg, Röntgenring 11, 97070 Würzburg, Germany.
** corresponding author (e-mail: lutz.nuhn@mpip-mainz.mpg.de).*

Material and Methods

Materials

Solvents and reagents were purchased and used without further purification unless described. 2,2-bis(hydroxymethyl)propionic acid (bis-MPA), triethylamine, benzyl bromide, ethyl chloro formate, trimethyl orthoisobutyrate, 1,2-diamino-*N,N'*-dimethylethane, rhodamine B octadecyl ester perchlorate, ammonium tetrafluoroborate, cesium fluoride, benzylamine, tetrahydrofuran (dry), methylene chloride (dry) and dimethylformamide (dry) were purchased from Sigma Aldrich.

For polymerization purposes, tetrahydrofuran was dried over sodium, distilled and stored in the glovebox. Analogously, methylene chloride was dried over calcium hydride.

Bis-(pentafluorophenyl)-carbonate and hexafluoroisopropanol (HFIP) were purchased from Fluorochem Ltd. and 5-(and-6-)-((N-(5-aminopentyl)amino)carbonyl)tetramethylrhodamine cadaverine (5(6)-TMR cadaverine) was purchased from Biotium. Poly(ethylene glycol) methyl ether ($mPEG_{44}$) was purchased from Rapp Polymere (Tübingen). Magnesium iodide (ultra dry) was purchased from Alfa Aesar in an ampoule and stored in a nitrogen-filled glovebox at -40 °C.

Millipore (mp) water was prepared using a MILLI-Q R Reference A+ System. Water was used at a resistivity of 18.2 MΩ·cm and total organic carbon of <5 ppm.

NMR spectroscopy

^{1}H, ^{19}F and DOSY NMR spectra were recorded on a Bruker Avance 300 MHz, a Bruker Avance 500 MHz spectrometer and a Bruker Avance III 700 MHz spectrometer. Diffusion ordered spectroscopy (DOSY) were conducted at room temperature and processed by Bayesian DOSY transformation (minimum: $1.00 \cdot 10^{-10}$, maximum: $1.00 \cdot 10^{-4}$, resolution factor: 1.00, repetition factor: 1, points in dimension: 128). Samples were prepared using deuterated solvents obtained from Sigma-Aldrich. Spectra were analyzed using MestReNova 14.2.0 by Mestrelab Research.

Size Exclusion Chromatography

Analytical size exclusion chromatography (SEC) was carried out at 40 °C on a SECcurity2instrument purchased from PSS, Mainz. Polymers were dissolved at a concentration of 1 mg/mL and injection volume was set to 30 µL. HFIP with 3 g/L potassium trifluoroacetate was used as an eluent and the flow rate was set at 0.8 mL/min. The column material was composed of modified silica gel (PFG columns, particle size: 7 µm, porosity: 100 Å+ 1000 Å), purchased from PSS Polymer Standards Service GmbH. For polymer detection a UV detector at a wavelength of λ = 254 nm/337 nm and a RI detector were employed. Furthermore, the system was equipped with a SECcurity2isocratic pump, a degasser, an auto sampler, and a column thermostat.

Molecular weights were determined by using a calibration with PMMA (PSS Polymer Standards Services GmbH). Evaluation of the elution diagram was conducted with PSS WinGPC from PSS Polymer Standard Service GmbH.

Dynamic Light Scattering

Dynamic light scattering measurements were performed using a Malvern Z Nano instrument equipped with a He-Ne-Laser (λ = 632.8 nm) using ZetaSizer Software 7.12. Measurements were performed in triplicates at 25 °C and a detection angle of 173°.

Ultraviolet-Vis Spectroscopy and Fluorescence Spectroscopy

UV-Vis spectra were recorded using a Thermo Scientific™ NanoDrop™ 2000c spectrophotometer. Brand UV micro cuvettes were used for the measurements.

Mass Spectrometry

MALDI measurements were carried out on a rapifleX™ MALDI-ToF/ToF mass spectrometer from Bruker Daltonik GmbH, Fahrenheitstraße 4, 28359 Bremen. The instrument is equipped with a scanning smartbeam 10 kHz Nd:YAG laser at a wavelength of 355 nm and a 10 bit 5 GHz digitizer. The acceleration voltage was set to 20 kV and the mass spectra were recorded in positive ion mode. Calibration was done by polymer standards of polyethylene glycol. Samples were prepared in DCM using a 2-[(2E)-3-(4-tert-butylphenyl)-2-methylprop-2-enylidene]malononitrile (DCTB) matrix.

Syntheses

Synthesis of NHO 1,3-dimethyl-2-(1-methylethylidene)imidazolidine

N-heterocyclic olefin was synthesized to a modified literature procedure[1] previously reported by Naumann et al.[2] By reaction of 1,2-diamino-*N,N'*-dimethylethane, trimethyl orthoisobutyrate and ammonium tetrafluoroborate, 1,3-dimethyl-2-(1-methylethyl)imidazolinium tetrafluoroborate salt was yielded. After addition of KH, the NHO 1,3-dimethyl-2-(1-methylethylidene)imidazolidine was liberated from the salt and stored in a glovebox at -40 °C.

Synthesis of 5-methyl-5-benzyloxycarbonyl-1,3-dioxan-2-one (MTC-OBn)

5-methyl-5-benzyloxycarbonyl-1,3-dioxane-2-one was synthesized by an adapted literature procedure.[3] In a first reaction step 2,2-bis(hydroxymethyl)propionic acid benzyl ester was yielded from the reaction of 2,2-bis(hydroxymethyl)propionic acid (bis-MPA) with benzyl bromide using the base potassium hydroxide.[4] Ring-closing reaction by ethyl chloro formate and triethylamine then gave 5-methyl-5-benzyloxycarbonyl-1,3-dioxan-2-one which was recrystallized several times from ethyl acetate to obtain colorless needles.[5] High purity was also previously demonstrated by X-ray diffraction.

^{1}H NMR (300 MHz, $CDCl_3$) δ [ppm] = 7.40–7.31 (5H, m, Aryl-*H*), 5.22 (2H, s, Aryl-CH_2), 4.70 (2H, d, CH_2), 4.20 (2H, d, CH_2), 1.33 (3H, s, CH_3).

Polymer synthesis of pyrene butanol-poly(MTC-OBn)

For the synthesis of pyrene butanol-poly(MTC-OBn), 0.1502 g MTC-OBn (0.600 mmol, 20 eq) and pyrene butanol (8.2 mg, 0.030 mmol, 1.0 eq.) was weighed into a 10 mL Schlenk-flask and dissolved in ~2 mL benzene. The mixture was freed from residual water by azeotropic removal of benzene and water overnight *in vacuo*. On the following day, the flask was transferred to a nitrogen-filled glovebox and freshly dried tetrahydrofuran (sodium dried) was added *via* syringe. Concentration was adjusted to 0.2 M (3 mL of THF) and the mixture of pyrene butanol and monomer was transferred to glass-oven dried screw lid glass (with a PTFE fitting and a magnetic stir bar). For ROP at -40 °C the mixtures were allowed to cool in the freezer for several hours before reaction was initiated by addition of 2.34 µL of NHO 1,3-dimethyl-2-(1-methylethylidene)imidazolidine (0.0022 mg, 0.015 mmol, 0.5 eq.) under stirring. For ROP at room temperature, reactions were initiated immediately by NHO addition. Conversion was checked regularly by ^{1}H NMR. For a degree of polymerization (DP) of 20 reaction time was 8 h at -40 °C. At room temperature after less than 2 h ~80-90% conversion was reached. Longer homopolymers with an aimed DP of 40 required reaction times of ~20 h at -40 °C. By addition of the polymerization mixture to a large excess of *n*-hexane a colorless solid precipitated and the mixture was centrifuged (4500 rpm, 10 min, 25 °C) and decanted. Subsequently, the pellet was dissolved in ~2 mL of THF and precipitated by dropwise addition to *n*-hexane. The procedure was repeated once and the product was then dried *in vacuo* over-night. Polymers were analyzed by HFIP SEC, NMR and MALDI-ToF.

^{1}H NMR (700 MHz, $CDCl_3$) δ [ppm] = 8.25–7.85 (9H, m, **a**), 7.35–7.26 (5H, m, **b**), 5.13 (2H, s, **c**), 4.27 (4H, s, **d**), 4.14 (2H, t, **e**), 3.37 (2H, t, **f**), 1.93 (2H, m, **g**), 1.82 (2H, m, **h**), 1.22 (3H, s, **i**).

HFIP SEC

RT pyrene butanol-poly(MTC-OBn)$_{20}$ M_n = 3120 g/mol, M_w = 5620 g/mol $Đ$ = 1.80 (RI detector)

-40 °C pyrene butanol-poly(MTC-OBn)$_{20}$ M_n = 4240 g/mol, M_w = 5470 g/mol $Đ$ = 1.29 (RI detector)

pyrene butanol-poly(MTC-OBn)$_{40}$ M_n = 8430 g/mol, M_w = 10200 g/mol $Đ$ = 1.21 (RI detector)

Polymer synthesis of mPEG$_{44}$-*b*-poly(MTC-OBn)

For the synthesis of amphiphilic block copolymers 0.0626 g MTC-OBn (0.250 mmol, 10 eq) was weighed into a 10 mL Schlenk-flask and dissolved in ~2 mL benzene. In another tube 0.050 g mPEG$_{44}$ (0.025 mol, 1 eq.) was dissolved in ~2 mL benzene (tube was shortly heated until PEG was fully dissolved). Both compounds were freed from residual water by azeotropic removal of benzene and water overnight *in vacuo*. Reaction was conducted in a nitrogen-filled glovebox at 0.2 M (1.25 mL of THF) catalyzed by NHO (0.0018 mg, 0.013 mmol, 0.5 eq.) at room temperature due to the limited solubility of mPEG$_{44}$ in THF at lower temperatures. After 45 minutes reaction time high conversion of ~84% was observed by NMR, reaction was stopped by precipitation in ice-cold diethylether (-20 °C), the mixture was centrifuged (4500 rpm, 10 min, 4 °C) and decanted. After two additional precipitation steps mPEG$_{44}$-*b*-poly(MTC-OBn) was yielded as a colorless powder. In analogous manner mPEG$_{44}$-*b*-poly(MTC-OBn)$_5$ was synthesized (0.100 g mPEG$_{44}$, 0.050 mol, 1.0 eq., and 0.0035 g NHO, 0.025 mmol, 0.5 eq.) with a conversion of ~89%.

^{1}H NMR (700 MHz, $CDCl_3$) δ [ppm] = 7.39–7.27 (5H, m, **a**), 5.12 (2H, s, **b**), 4.27 (4H, s, **c**), 3.63 (4H, s, **d**), 3.36 (3H, s, **e**), 1.22 (3H, s, **f**).

HFIP SEC mPEG$_{44}$-*b*-poly(MTC-OBn)$_4$ M_n = 19290 g/mol, M_w = 23370 g/mol $Đ$ = 1.21 (RI detector)

mPEG$_{44}$-*b*-poly(MTC-OBn)$_7$ M_n = 19240 g/mol, M_w = 21760 g/mol $Đ$ = 1.13 (RI detector)

Synthesis of 5-methyl-5-pentafluorophenyloxycarbonyl-1,3-dioxane-2-one (MTC-PFP)

5-Methyl-5-pentafluorophenyloxycarbonyl-1,3-dioxane-2-one (MTC-PFP) was synthesized by cyclization of 2,2-bis(hydroxymethyl)propionic acid (bis-MPA) using bis-(pentafluorophenyl)-carbonate as reported in the literature.[6] The crude product was recrystallized two times from a 1:1 mixture of ethyl acetate with hexane to yield colorless crystals.

^{1}H NMR (300 MHz, $CDCl_3$) δ = 4.85 (d, ^{2}J(H,H)) = 11 Hz, 2H; CH_2), 4.35 (d, ^{2}J(H,H), 2 H; CH_2) = 11 Hz, 2H), 1.56 (s, 3H, CH_3).

^{19}F NMR (376 MHz, $CDCl_3$) δ [ppm] = -153.71 – -154.23 (d, ^{3}J(F,F) = 22 Hz, 2 F; *m*-F), -157.13 (dd, ^{3}J(F,F) = 22 Hz, 1 F; *p*-F), -162.26 (dd, ^{3}J(F,F) = 22 Hz, 2 F; *o*-F).

NHO-catalyzed transesterification of 5-methyl-5-pentafluorophenyloxycarbonyl-1,3-dioxane-2-one (MTC-PFP) by tetraethylene glycol monomethyl ether

Transesterification of MTC-PFP (0.0489 g, 0.150 mmol, 1.0 eq.) was performed using tetraethylene glycol monomethyl ether (0.0312 g, 0.150 mmol, 1.0 eq.) under NHO catalysis. MTC-PFP and tetraethylene glycol monomethyl ether were independently dried by distillation with benzene under reduced pressure. Reaction was conducted at room temperature under inert atmosphere using 3.51 µL NHO (0.0032 g, 0.023 mmol, 0.15 eq.) as catalyst. ^{1}H and ^{19}F NMR of the reaction mixture showed ~88% conversion after 40 h and the formation of the transesterification product MTC-OEG_4.

Polymer synthesis of pyrene butanol-poly(MTC-PFP)

For the synthesis of pyrene butanol-poly(MTC-PFP), 0.0978 g MTC-OBn (0.300 mmol, 20 eq) and pyrene butanol (4.1 mg, 0.015 mmol, 1.0 eq.) was weighed into a 10 mL Schlenk-flask and dissolved in ~2 mL benzene. The mixture was freed from residual water by azeotropic removal of benzene and water overnight *in vacuo*. On the following day, the flask was put into a nitrogen-filled glovebox, the mixture was dissolved in freshly dried THF (1.5 mL) and transferred by syringe to a glass-oven dried screw lid glass. In another screw lid glass 1.5 mL of dry THF was added to magnesium iodide (6.3 mg, 0.023 mmol, 1.5 eq.) and stirred for several hours to dissolve the salt. Both mixtures were then allowed to cool to -20 °C (lower temperatures of - 40 °C could not be applied due to the limited solubility of MTC-PFP in THF). Then both mixtures were combined and reaction was initiated by addition of 2.34 µL NHO (2.1 mg, 0.015 mmol, 1.0 eq.). Conversion was regularly checked by ^{1}H and ^{19}F NMR. For a degree of polymerization (DP) of 20, reaction times of 2-4 h were required to reach conversion of ~80 % (-20 °C). Longer homopolymers with an aimed DP of 40 required reaction times of ~20 h. Polymerization mixtures were precipitated by addition to a large excess of *n*-hexane and handled in an analogous manner as described above for MTC-OBn homopolymers, yielding slight yellow products. For salt removal, polymers were dissolved in THF and filtered over a short silica gel column (ethyl acetate as eluent) resulting in colorless products. Polymers were dried *in vacuo* over-night and analyzed by HFIP SEC, NMR (^{1}H, ^{19}F) and MALDI-ToF.

^{1}H NMR (700 MHz, $CDCl_3$) δ [ppm] = 8.25–7.85 (9H, m, **a**), 4.45 (4H, s, **b**), 4.23 (2H, t, **c**), 3.38 (2H, t, **d**), 1.95 (2H, m, **e**), 1.87 (2H, m, **f**), 1.48 (3H, s, **g**).

HFIP SEC

pyrene butanol-poly(MTC-PFP)$_{17}$ M_n = 2760 g/mol, M_w = 3490 g/mol $Đ$ = 1.24 (RI detector)

pyrene butanol-poly(MTC-PFP)$_{34}$ M_n = 5460 g/mol, M_w = 6820 g/mol $Đ$ = 1.25 (RI detector)

Polymer synthesis of mPEG$_{44}$-*b*-poly(MTC-PFP)

For the synthesis of reactive precursor block copolymers 0.0626 g MTC-PFP (0.250 mmol, 5 eq) was weighed into a 10 mL Schlenk-flask and dissolved in ~2 mL benzene. In another tube 0.050 g mPEG$_{44}$ (0.025 mol, 1 eq.) was dissolved in ~2 mL benzene. Both compounds were freed from residual water by azeotropic removal of benzene and water overnight *in vacuo*. Reaction was conducted in a nitrogen-filled glovebox at 0.1 M (2.5 mL of THF) catalyzed by NHO (0.0035 mg, 0.025 mmol, 1.0 eq.) and magnesium iodide (0.0104 mg, 0.0375 mmol, 1.5 eq.) at room temperature. After 4 h reaction time high conversion of ~64% was observed by ^{1}H and ^{19}F NMR, reaction was stopped by precipitation in ice-cold diethylether (-20 °C), the mixture was centrifuged (4500 rpm, 10 min, 4 °C) and decanted. After two additional precipitation steps mPEG$_{44}$-*b*-poly(MTC-PFP) was yielded as a colorless powder. In analogous manner mPEG$_{44}$-*b*-poly(MTC-PFP)$_5$ was synthesized (0.100 g mPEG$_{44}$, 0.050 mol, 1.0 eq., 0.0070 g NHO, 0.050 mmol, 1.0 eq., and 0.0209 mg magnesium iodide, 0.075 mmol, 1.5 eq.) with a conversion of ~75% after 2 h.

^{1}H NMR (700 MHz, $CDCl_3$) δ [ppm] = 4.46 (s, 4H, **a**), 3.63 (s, 4H, **b**), 3.36 (s, 3H, **c**), 1.49 (s, 3H, **d**).

HFIP SEC mPEG$_{44}$-*b*-poly(MTC-PFP)$_5$ M_n = 19290 g/mol, M_w = 22500 g/mol $Đ$ = 1.17 (RI detector)

mPEG$_{44}$-*b*-poly(MTC-PFP)$_7$ M_n = 19440 g/mol, M_w = 22070 g/mol $Đ$ = 1.14 (RI detector)

Synthesis of dye-labeled mPEG$_{44}$-*b*-poly(MTC-NHBn) block copolymers by post functionalization

For postmodification 0.0100 g of reactive precursor polymer mPEG$_{44}$-*b*-poly(MTC-PFP)$_7$ (2.283 µmol of polymer chains containing 16.666 µmol PFP ester units, 1.0 eq.) was dissolved in 1 mL of dry DMF and 2.48 µL triethylamine (0.0018 g, 17.902 µmol, 1.30 eq. related to the amount of PFP ester units) was added. First TMR-cadaverine (17.2 µL of 5 mg/mL solution in DMSO, 0.167 µmol, 0.25 eq., ~1 wt% of dye) was conjugated to the polymer (25 min reaction time) and then remaining PFP esters were converted with 3.08 µL of benzyl amine (0.00302 g, 21.662 µmol, 1.30 eq., 25 min reaction time). Polymers were then precipitated by addition of the mixture to 10 mL of diethylether (-20 °C) yielding a pink solid, the colorless diethyl ether supernatant indicated successful and complete dye labeling. For particle assembly the solid was dissolved in 1 mL DMSO and dialyzed against diluted hydrochloric acid (pH 4) in a 1 kDa dialysis bag with frequent exchange of the medium. After freeze drying samples were assembled by solvent-evaporation method and analyzed by DLS and UV-Vis confirming successful particle formation and dye labeling. Analogously a dye-labeled block copolymer was yielded from mPEG$_{44}$-*b*-poly(MTC-PFP)$_5$. Micellar self-assembly could be achieved similarly to mPEG$_{44}$-*b*-poly(MTC-oBn).

Further Experiments

Assembly and dye-loading of polymeric micelles by solvent-evaporation

Micellar assembly of mPEG$_{44}$-*b*-poly(MTC-OBn) and mPEG$_{44}$-*b*-poly(MTC-NHBn) was achieved by solvent-evaporation method as described previously.[3] Briefly, polymers (2.5 mg) were dissolved in acetone at 5 mg/mL and for mPEG$_{44}$-*b*-poly(MTC-OBn) the respective amount of hydrophobic dye rhodamine B octadecyl ester perchlorate was added (1 wt%) from an acetone stock solution. Mixtures were then dropped into mp-water and gave a 5 mg/mL aqueous solution after evaporation of acetone over-night. To filter off excess dye, samples were filtered using a syringe filter with a pore size of 0.22 µm (membrane material: hydrophilized PTFE). Characterization by DLS and UV-Vis confirmed micelle formation and dye-loading.

Determination of the effects of organocatalysts on the metabolic activity by MTT Assay using Raw Blue macrophages

For the analysis of the cell's metabolic activity as an indicator for toxicity and cell proliferation, Raw Blue macrophages were seeded into 96-well plates (90000 cells/well in 180 µL culture medium) and incubated with 20 µL of organocatalyst solutions. Concentrations were ranging from 0.4 µM to 400 µM (equal 0.056 µg/mL to 0.056 mg/mL for NHO and 0.060 µg/mL to 0.060 mg/mL for trifluoromethanesulfonic acid, TFMSA). On the next day (after 24 h) 30 µL of 2 mg/mL 3-(4,5-dimethylthiazol-2-yl)-2,5-diphenyltetrazolium bromide solution in phosphate buffered saline (PBS) was added. After 3 h of incubation time, formazan crystals were dissolved by the addition of 100 µL of 10% m/v SDS/0.01 M HCl and incubated overnight at 37 °C. Absorbance was measured at 590 nm using a plate reader. All experiments were performed in quadruplicates.

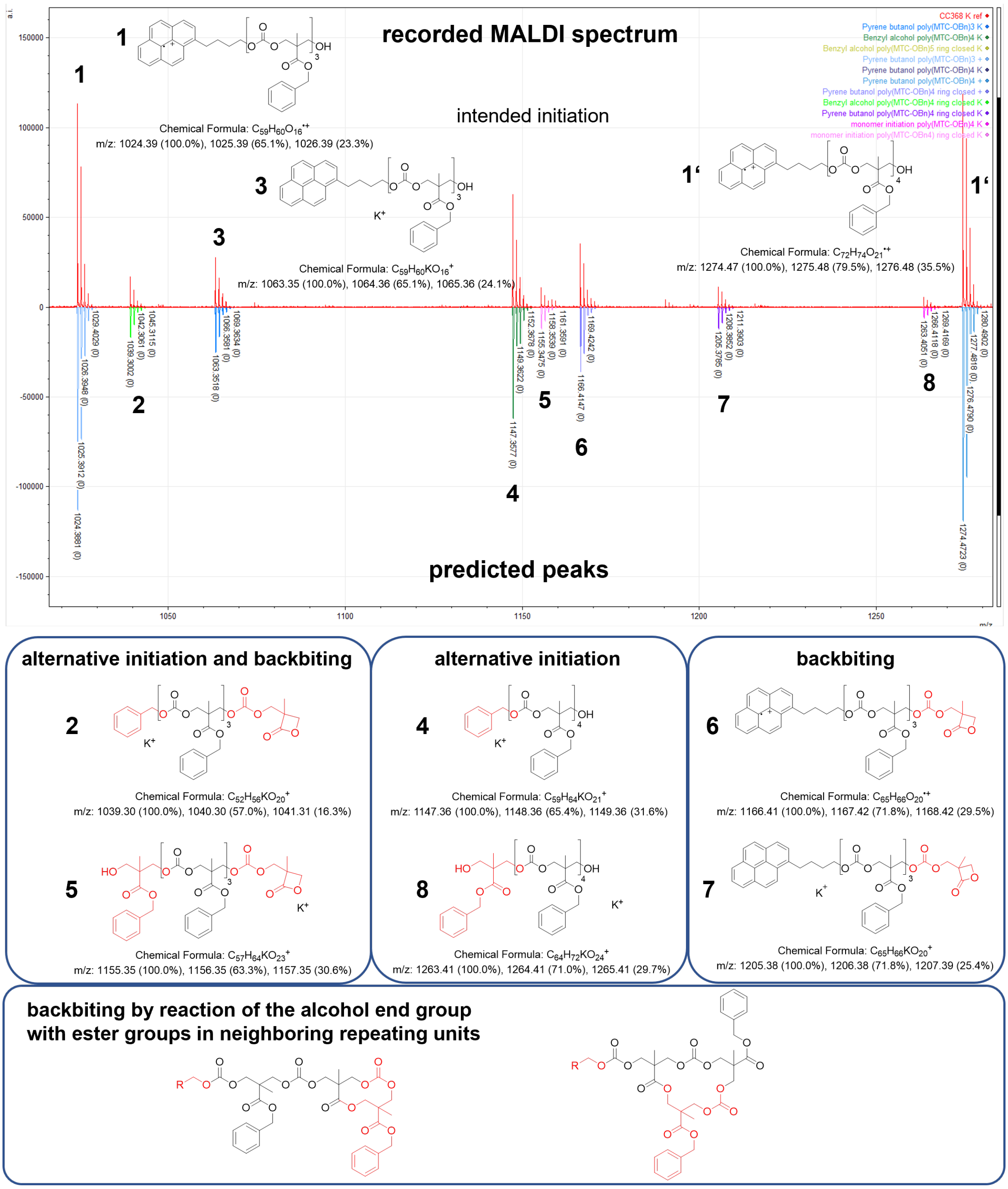

Figure S 1 Detailed MALDI-ToF analysis of polymerization products of the uncontrolled polymerization of MTC-OBn catalyzed by NHO at room temperature (view ranging from 1020-1275 m/z). Polymers with the intended initiation and end groups (**1**¸**3** and **1’**) were identified and found with highest intensities. In addition to K^+ species (**3**) pyrene containing species show a radical cation species (**1**) that results from photoionization of the pyrene moieties. Arising side reactions at suboptimal reaction conditions (+25 °C) yield additional products: Backbiting reactions resulting in benzyl alcohol release and the formation of ring-closed end groups by a 4-exo-trig ring closure (right box, **6** and **7**). Thereby, 4-ring lactones can be formed (as depicted) or backbiting takes place at side-groups of neighboring repeating units, which results in the formation of larger ring-systems (depicted on the bottom for backbiting in neighboring repeating units). Released benzyl alcohol then results in alternative initiation sites (middle box, **4** and **8**). Polymer **8** is most likely formed by monomer initiation but might also be yielded by backbiting or chain reshuffling reactions at the carbonate motif. These polymers (**4** and **8**) can also undergo backbiting reactions (left box) forming polymer chains with alternative initiation and backbiting (**2** and **5**).

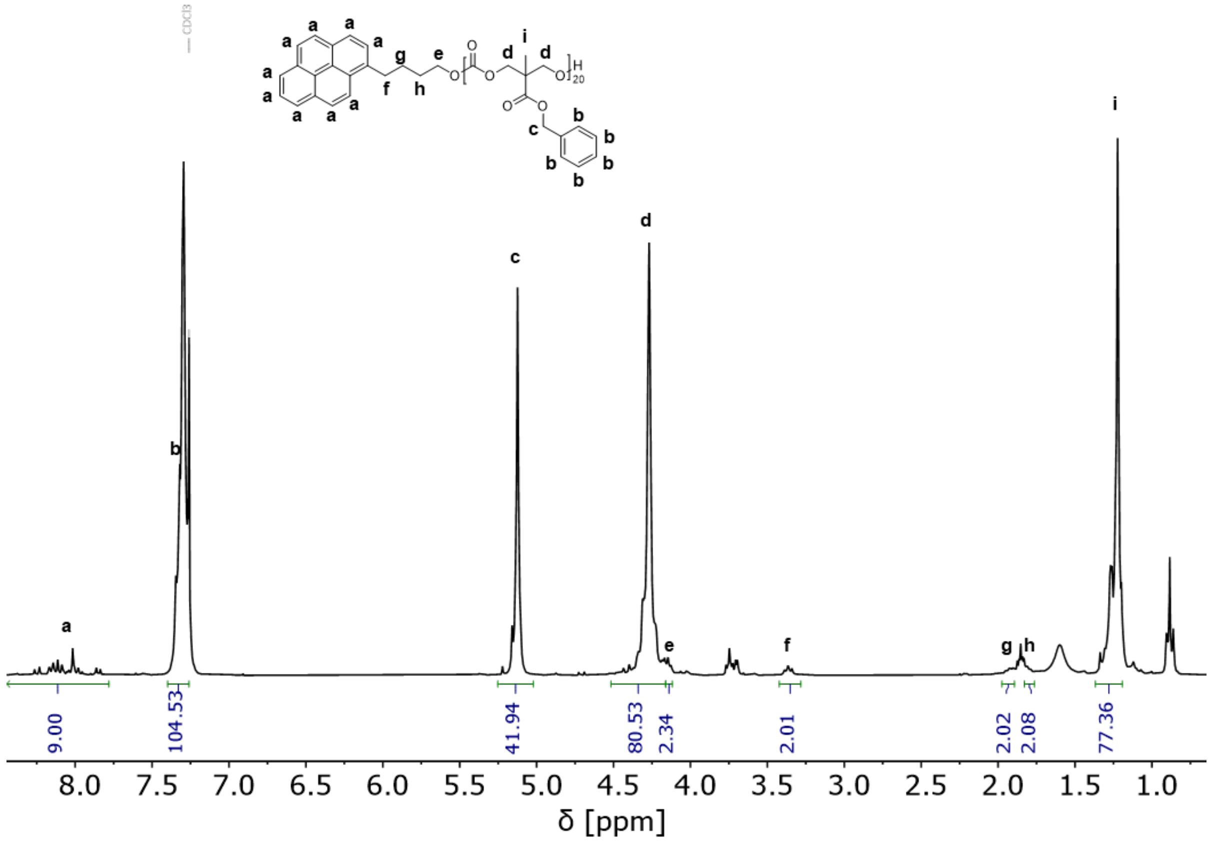

Figure S 2 ^{1}H NMR of pyrene butanol-poly(MTC-OBn)$_{20}$ prepared by uncontrolled ROP at +25 °C. Dissolved in $CDCl_3$ for NMR measurements.

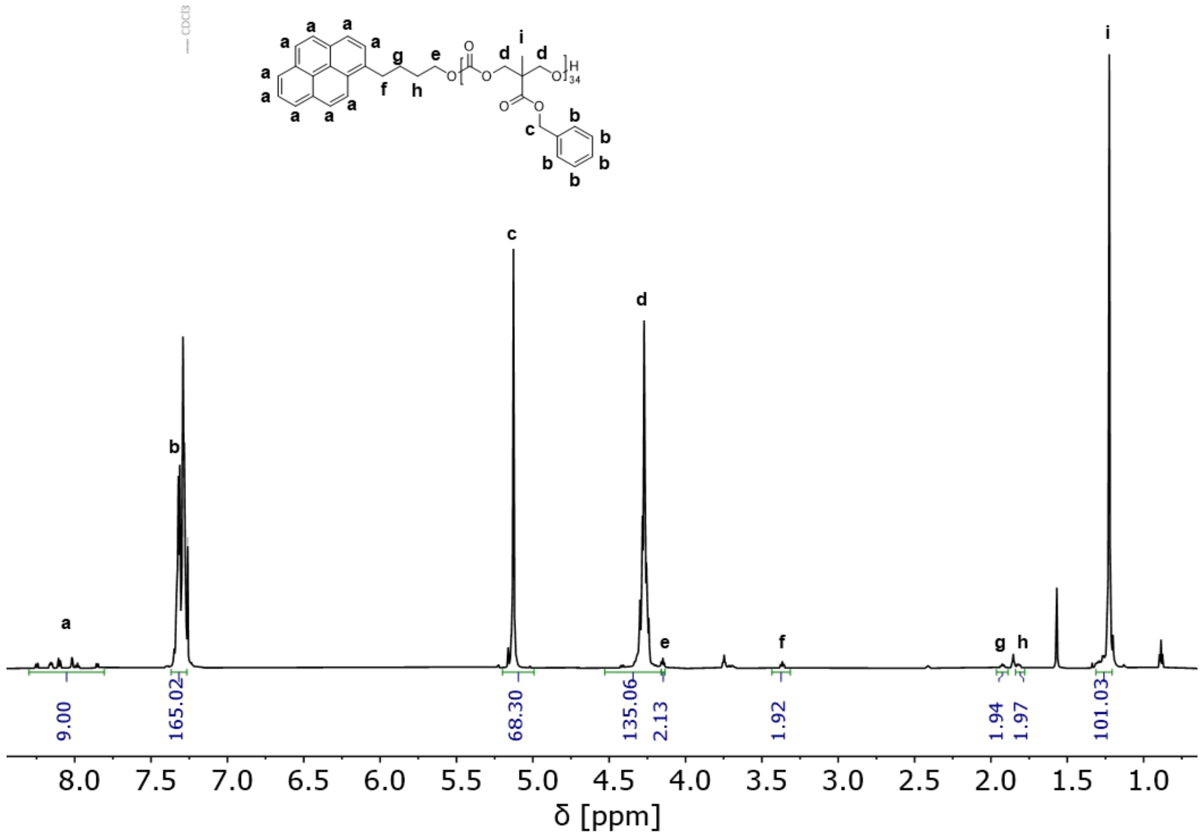

Figure S 3 ^{1}H NMR spectrum of pyrene butanol-poly(MTC-OBn)$_{34}$ prepared by controlled ROP at -40 °C in $CDCl_3$.

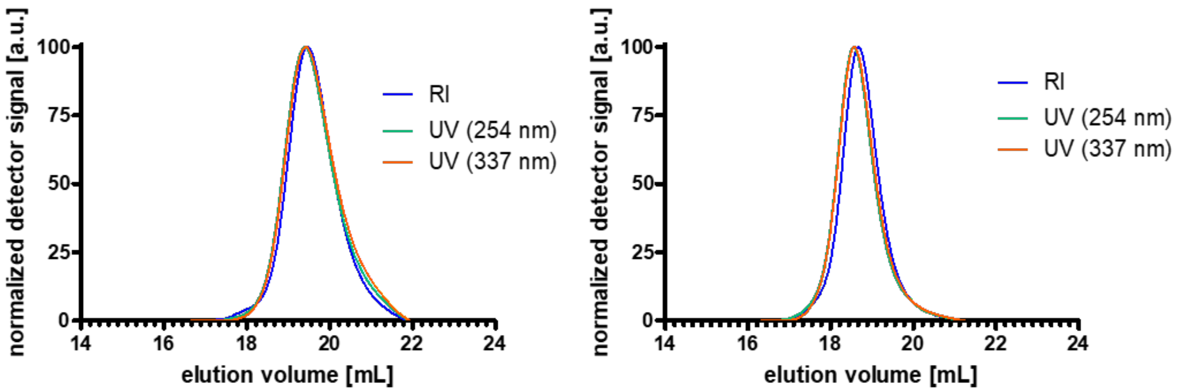

Figure S 4 Additional SEC traces of MTC-OBn homopolymers prepared by controlled ROP at -40 °C showing RI, UV (254 nm) and UV traces (337 nm) for pyrene butanol-poly(MTC-OBn)$_{18}$ (left) and pyrene butanol-poly(MTC-OBn)$_{34}$ (right).

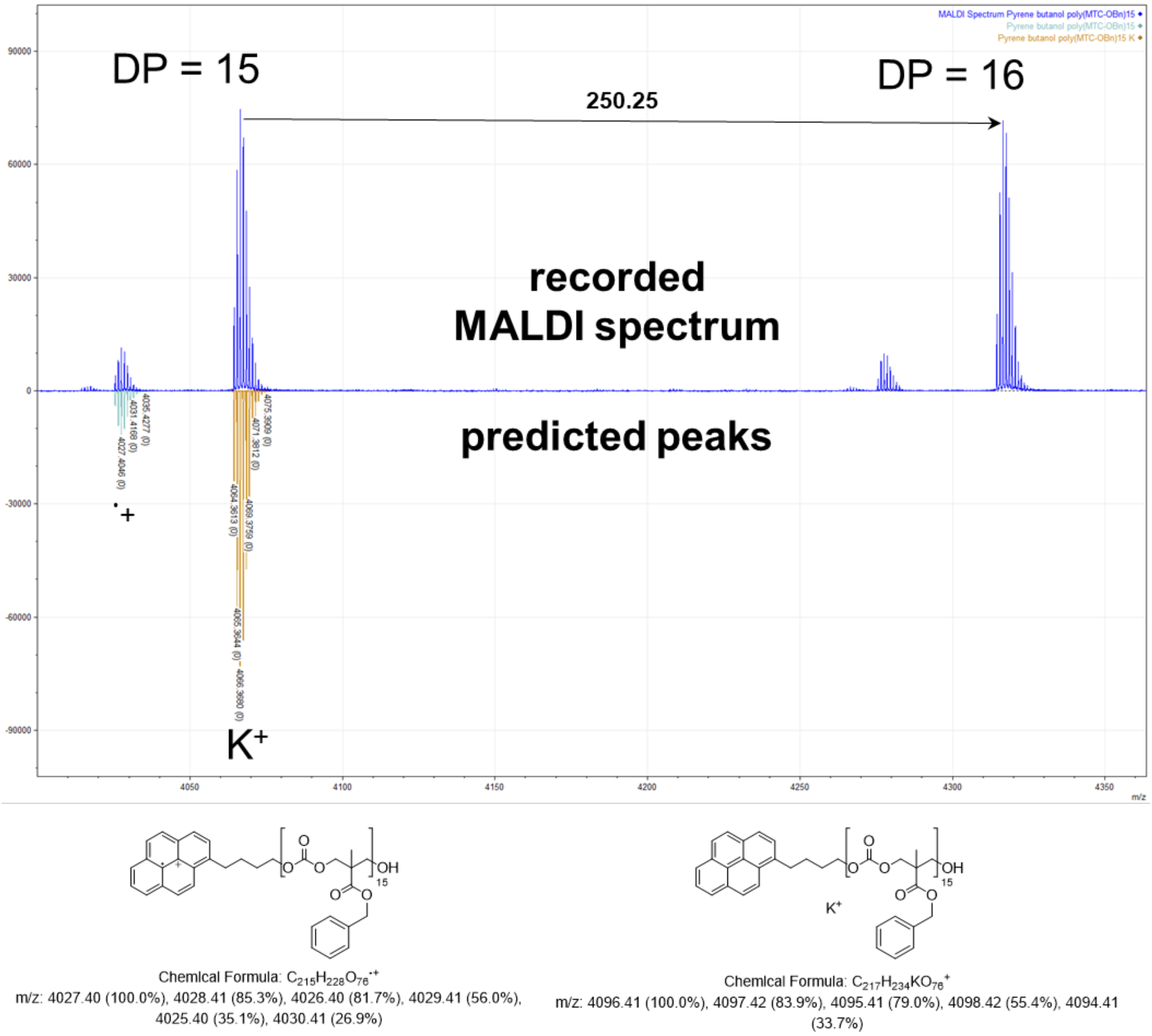

Figure S 5 Detailed MALDI-ToF analysis of polymerization products of the controlled ROP of MTC-OBn catalyzed by NHO at decreased temperature (-40 °C, view at highest intensity of the spectrum ranging from 4000-4350 m/z). There are only species found with the intended initiator (pyrene butanol) and end groups (hydroxy). Cationization by potassium K$^+$ as well as radical cation formation (·+) by photoionization of the pyrene end group are found simultaneously. No further side products are detected, proving the highly defined nature of NHO-catalyzed at -40 °C.

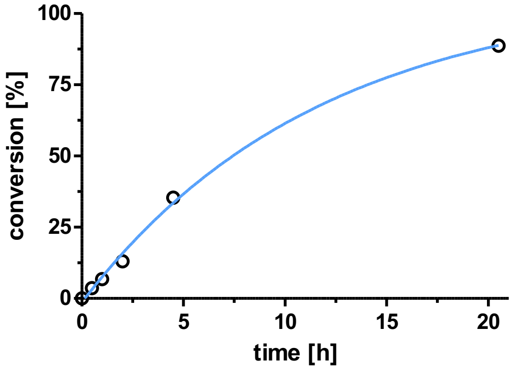

Figure S 6 Conversion of MTC-OBn polymerization (targeted DP = 40) initiated by pyrene butanol as analyzed by ^{1}H NMR.

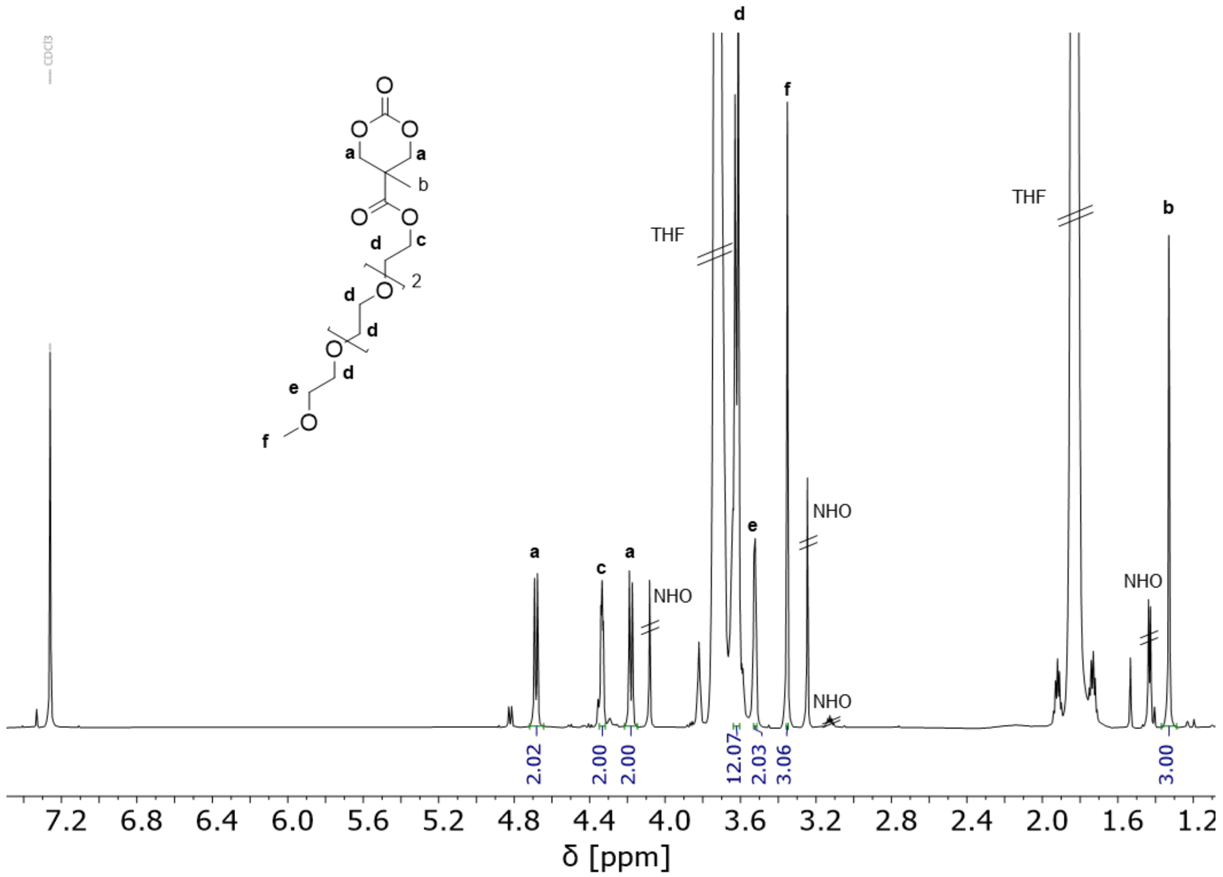

Figure S 7 ^{1}H NMR spectrum of MTC-OEG$_4$ ($CDCl_3$) after transesterification of MTC-PFP with tetraethylene glycol monomethyl ether by NHO catalysis.

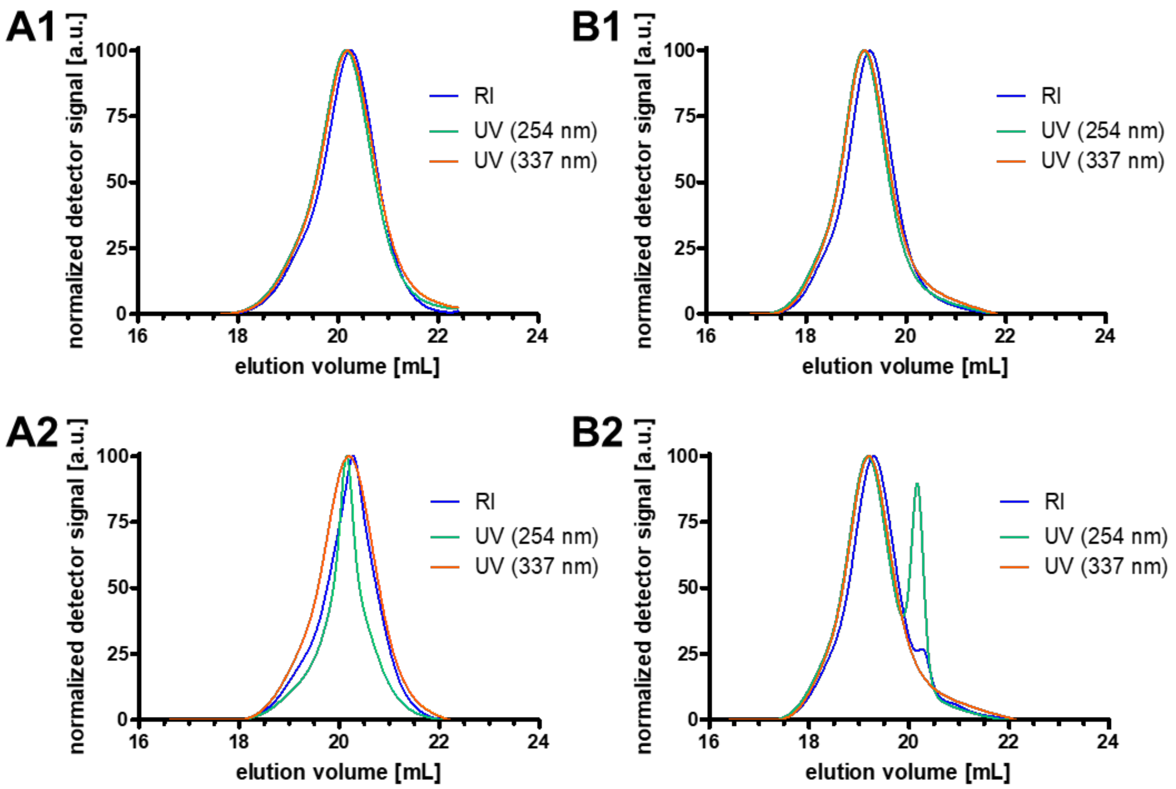

Figure S 8 HFIP SEC traces of pyrene butanol-poly(MTC-PFP)$_{17}$ (A1+2) and pyrene butanol-poly(MTC-PFP)$_{29}$ (B1+2). Polymers after removal of NHO-salt complex are depicted in the top row (A1+B1). In the bottom row (A2+B2), respective polymers are shown before removal of the NHO-salt complex (strong signal in UV at ~19.5 mL elution volume) by silica column. Overlap of RI traces before and after purification prove that polymer samples are unaltered by column purification.

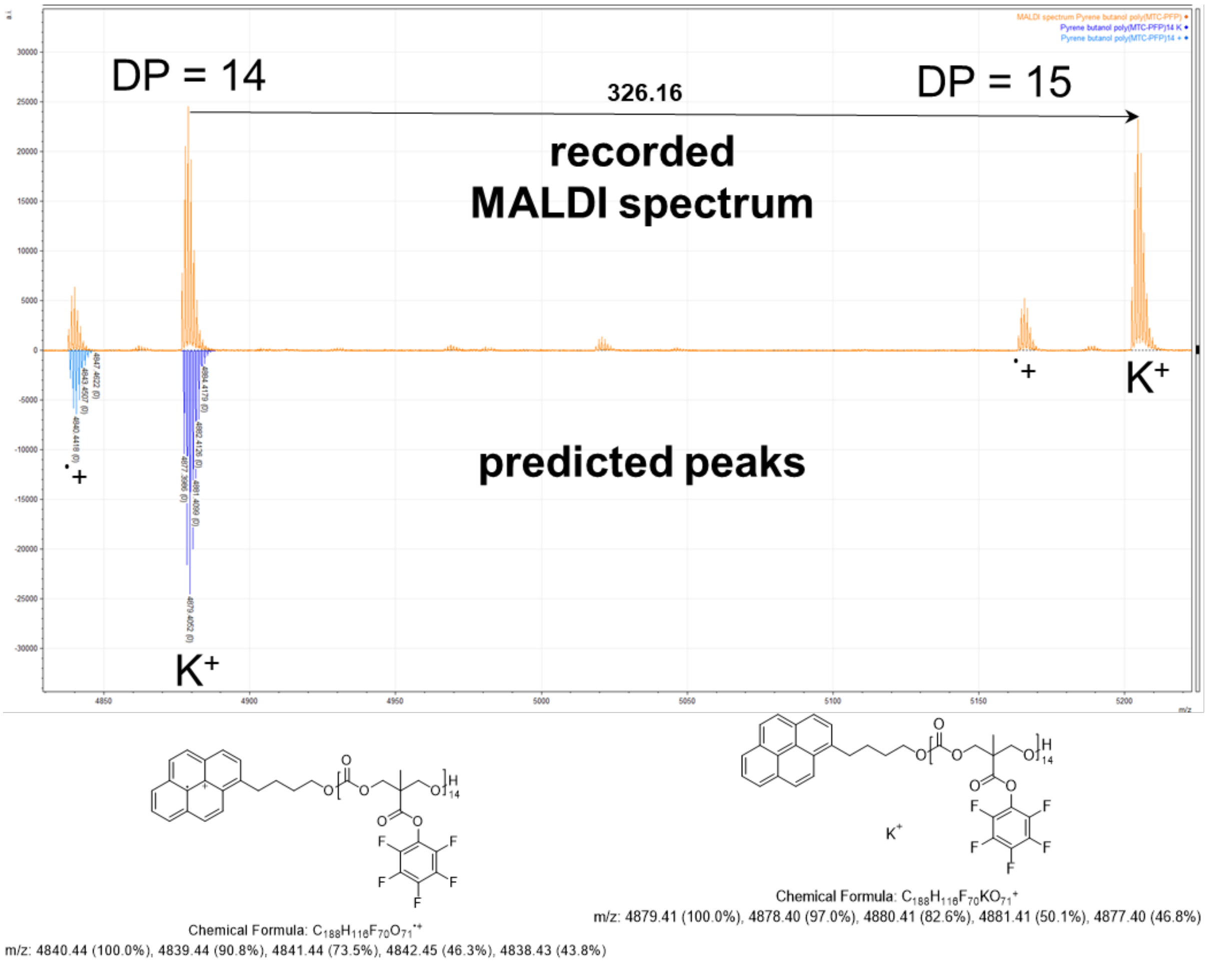

Figure S 9 Detailed MALDI-ToF analysis of polymerization products of the controlled ROP of MTC-PFP catalyzed by cocatalysis at decreased temperature (-20 °C). Products show the intended initiator (pyrene butanol) and end groups (hydroxy). Cationization by potassium K^+ as well as radical cation formation (·+) by photoionization of the pyrene end group are found simultaneously (also a minor distribution cationized with Na^+ is found) proving the highly defined nature of cocatalysis at -20 °C.

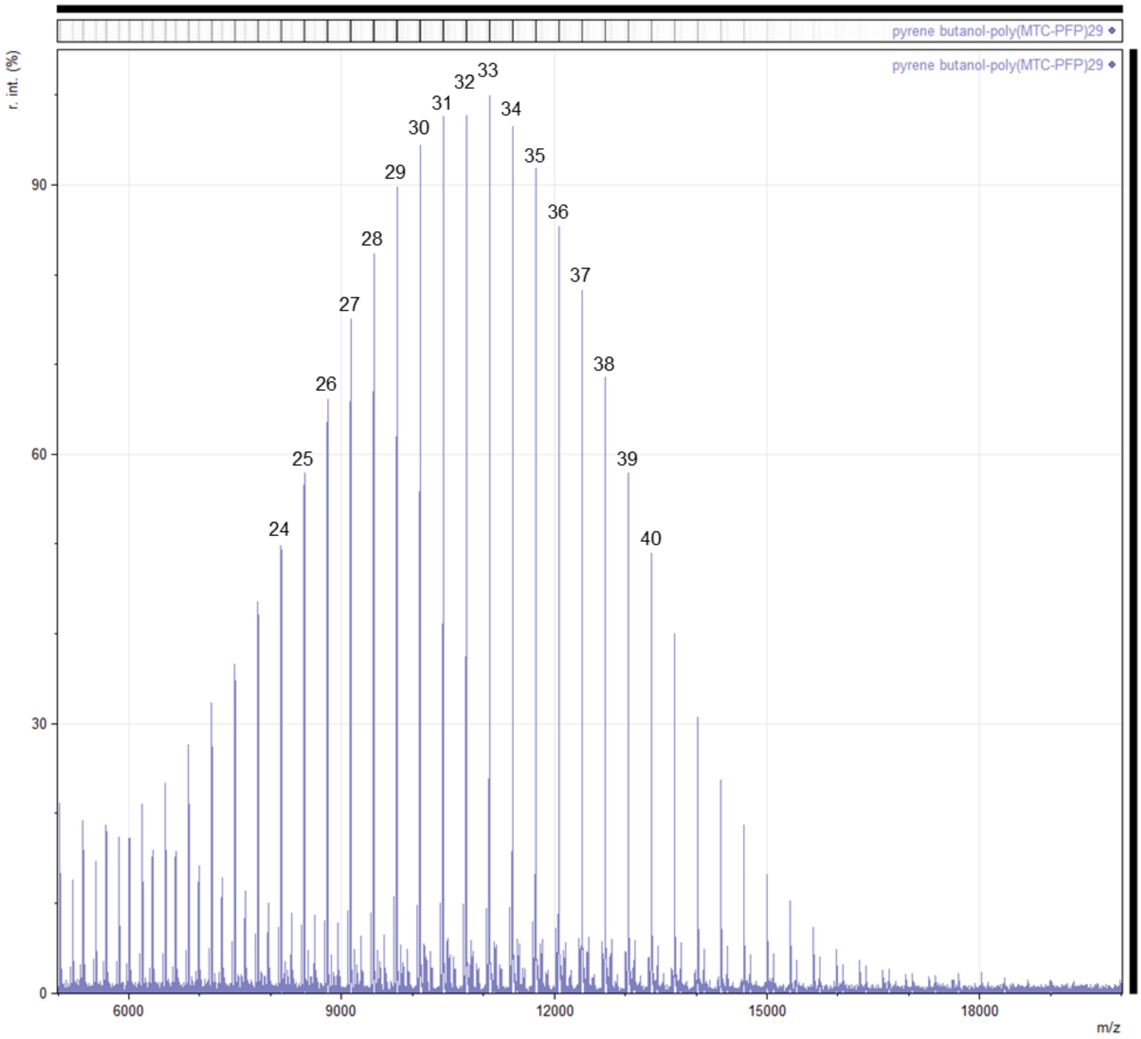

Figure S 10 MALDI-ToF spectrum of pyrene butanol-poly(MTC-PFP)$_{29}$ showing pyrene butanol-poly(MTC-PFP) cationized by K^+ and radical cation formation by photoionization (·+) with the assigned degrees of polymerization.

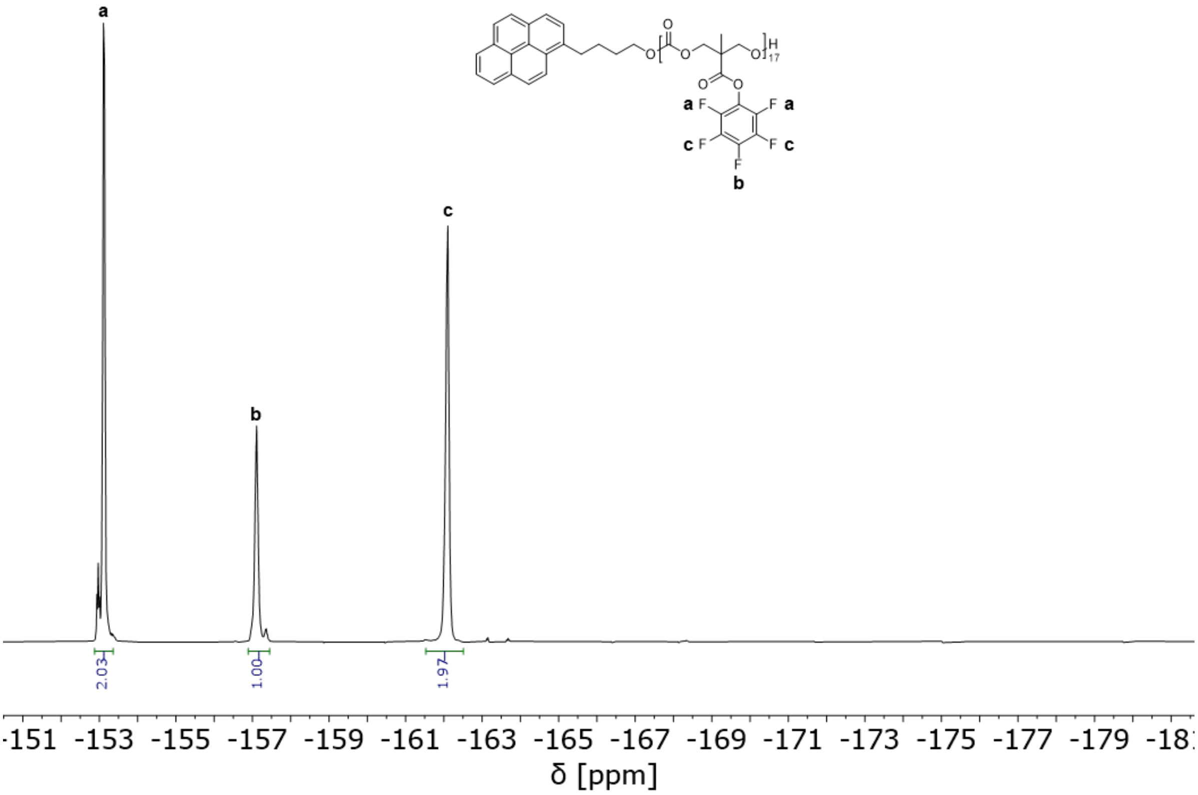

Figure S 11 ^{19}F NMR spectrum of pyrene butanol-poly(MTC-PFP)$_{17}$ in $CDCl_3$.

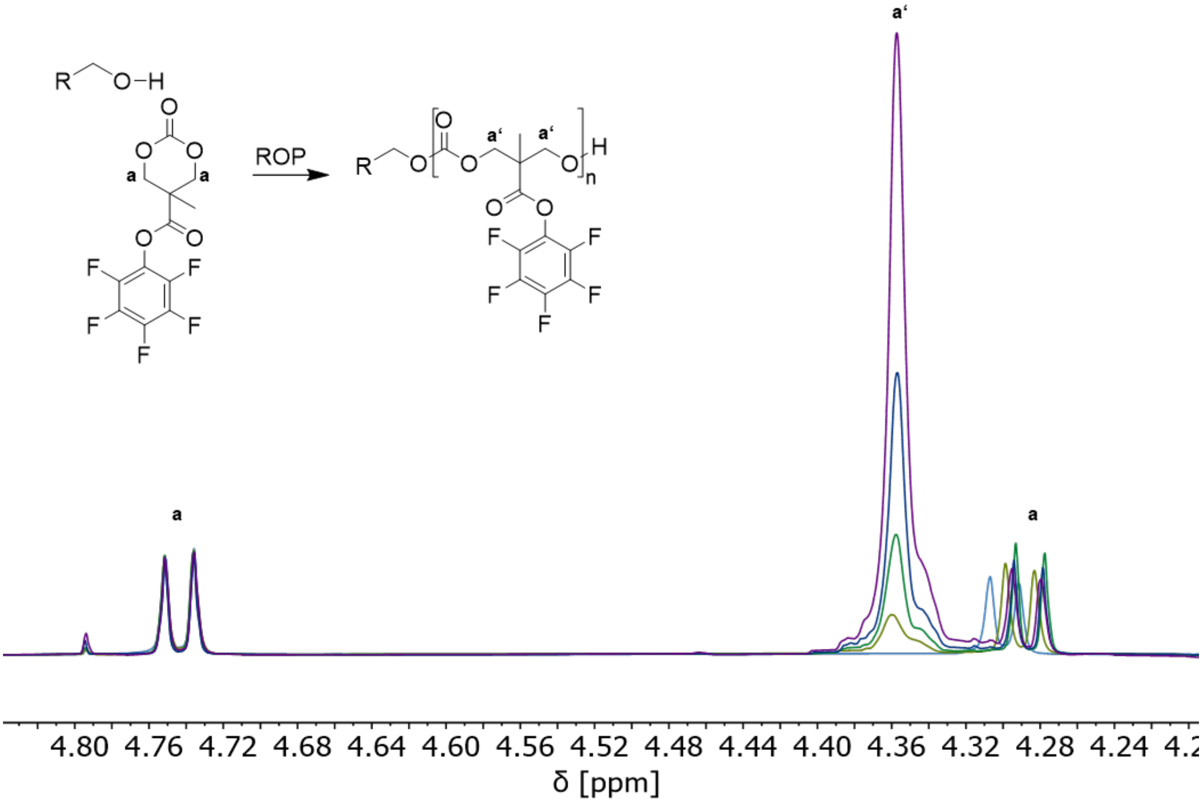

Figure S 12 ^{1}H NMR spectrum of cocatalyzed ROP of MTC-PFP at various time points (0 min, 15min, 30 min, 1 h, 2 h) showing the polymerization progress by increasing polymer signal **a'** and (relative) decline of monomer signal **a**.

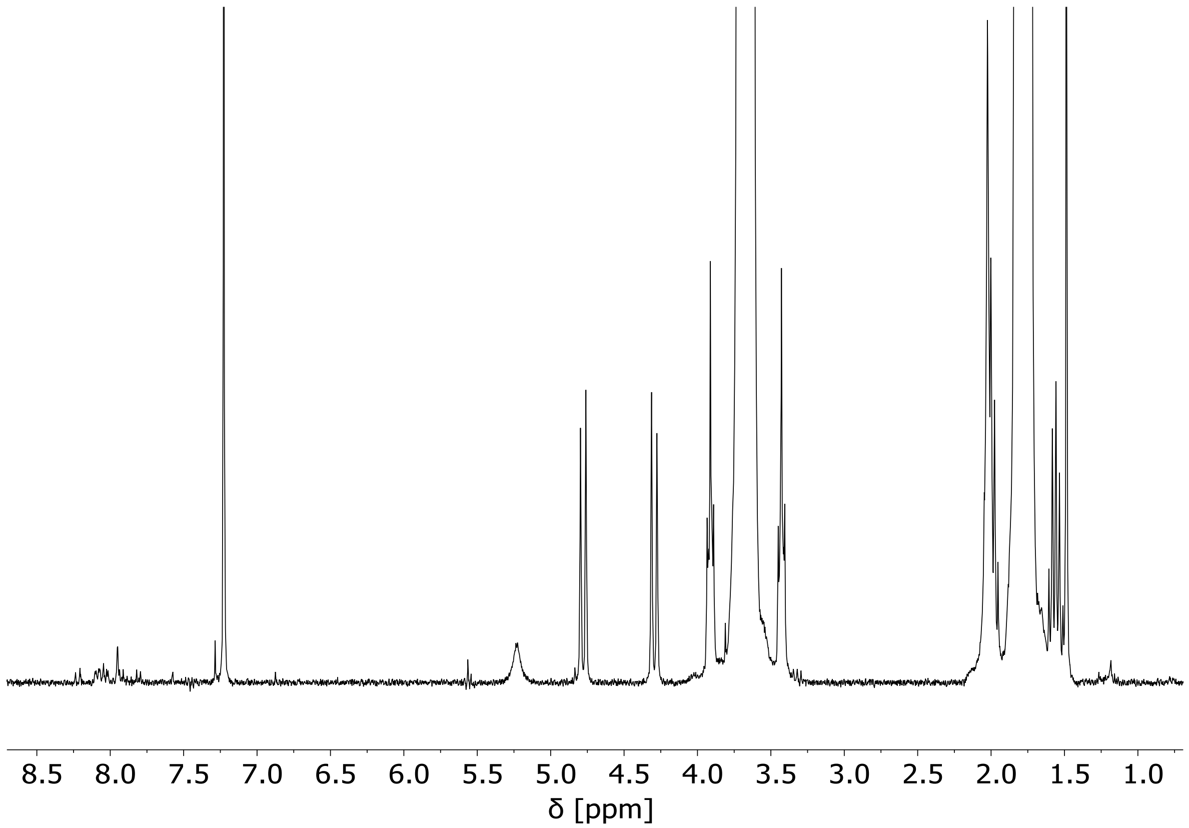

Figure S 13 ^{1}H NMR spectrum of MTC-PFP/pyrene butanol mixture when exposed to MgI_2 after 5 d at -20 °C. Neither polymerization nor the transesterification of active ester substituents is observed, proving that the sole action of MgI_2 is insufficient to trigger polymerization or transesterification of MTC-PFP and that indeed cocatalysis of NHO and MgI_2 is required for ROP of MTC-PFP.

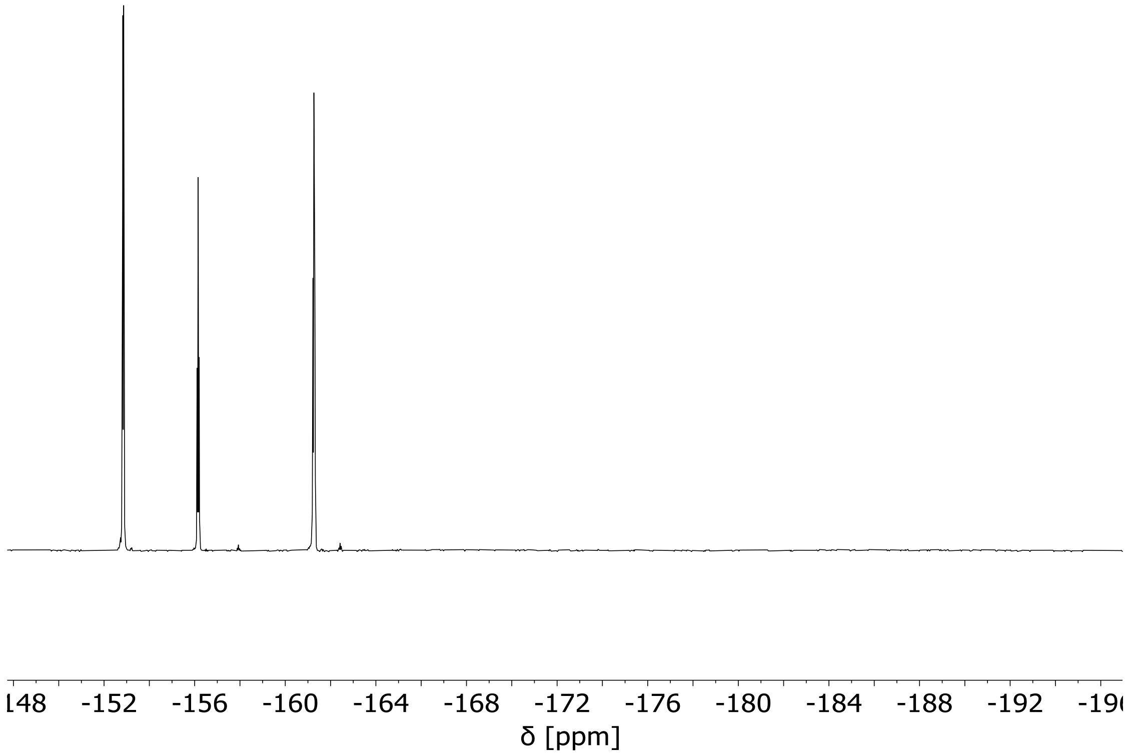

Figure S 14 ^{19}F NMR spectrum of MTC-PFP/pyrene butanol mixture when exposed to MgI_2 after 5 d at -20 °C. Signals of MTC-PFP ester group is unaltered, showing the stability of the active ester against Lewis acid MgI_2 as well as that polymeric species are not formed by the sole action of MgI_2. Both reactions would result in additional ^{19}F species as shown in previous figures.

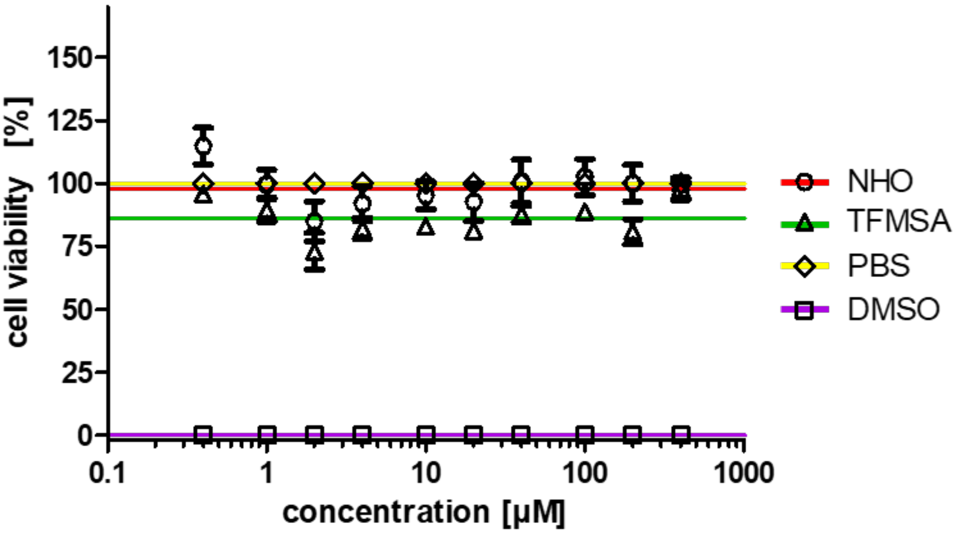

Figure S 15 MTT assay of organocatalyst 1,3-dimethyl-2-(1-methylethylidene)imidazolidine (NHO, red line) and trifluoromethane sulfonic acid (TFMSA, green line). Both organocatalysts showed no adverse effects on the viability of raw blue macrophages after incubation for 20 h with concentrations ranging from 0.4 µM to 400 µM (equal 0.056 µg/mL to 0.056 mg/mL for NHO and 0.060 µg/mL to 0.060 mg/mL for TFMSA). PBS and DMSO are shown as a positive and negative control.

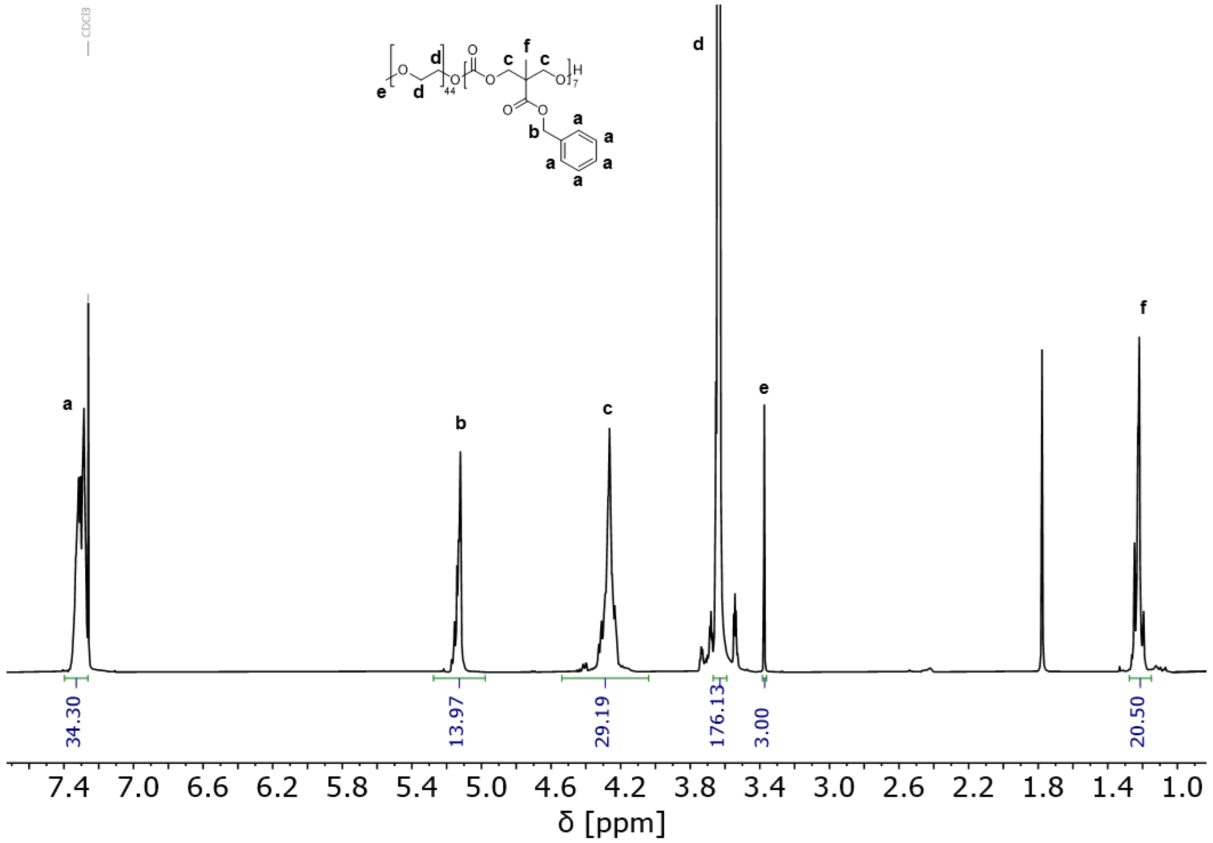

Figure S 16 ^{1}H NMR spectrum of mPEG$_{44}$-*b*-poly(MTC-OBn)$_7$ in CDCl$_3$.

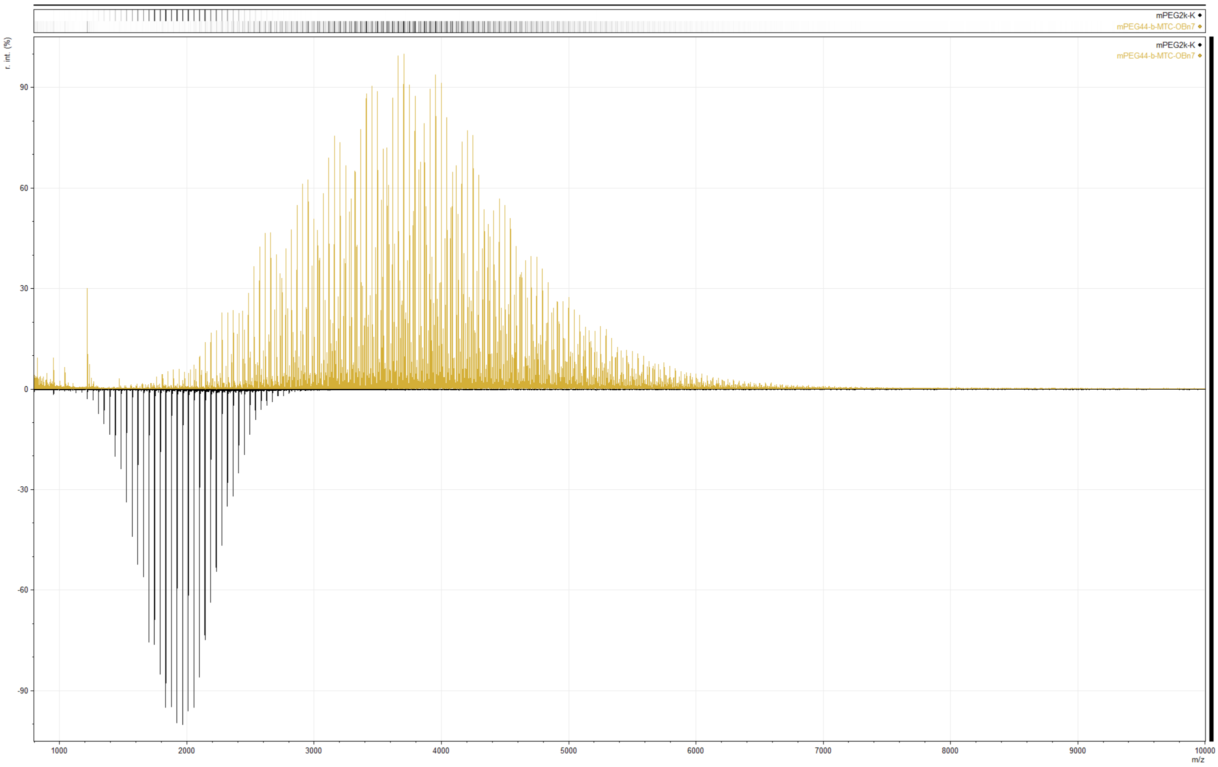

Figure S 17 MALDI-ToF spectra of mPEG$_{44}$-*b*-poly(MTC-OBn)$_7$ (top, yellow spectrum) and mPEG$_{44}$ (bottom, black spectrum). MALDI-ToF shows the successful polymerization of the polycarbonate block onto mPEG$_{44}$ and a narrowly distributed spectrum.

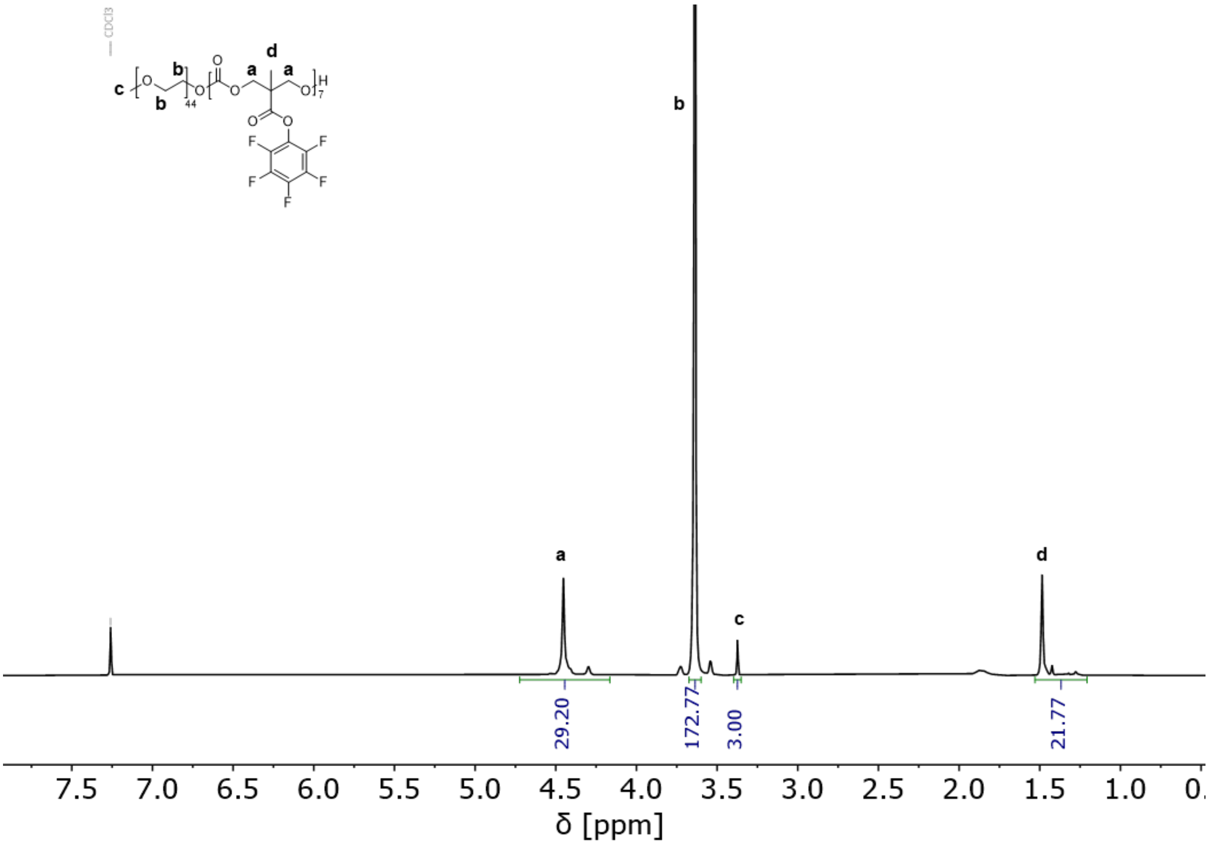

Figure S 18 ^{1}H NMR spectrum of mPEG$_{44}$-*b*-poly(MTC-PFP)$_7$ in $CDCl_3$.

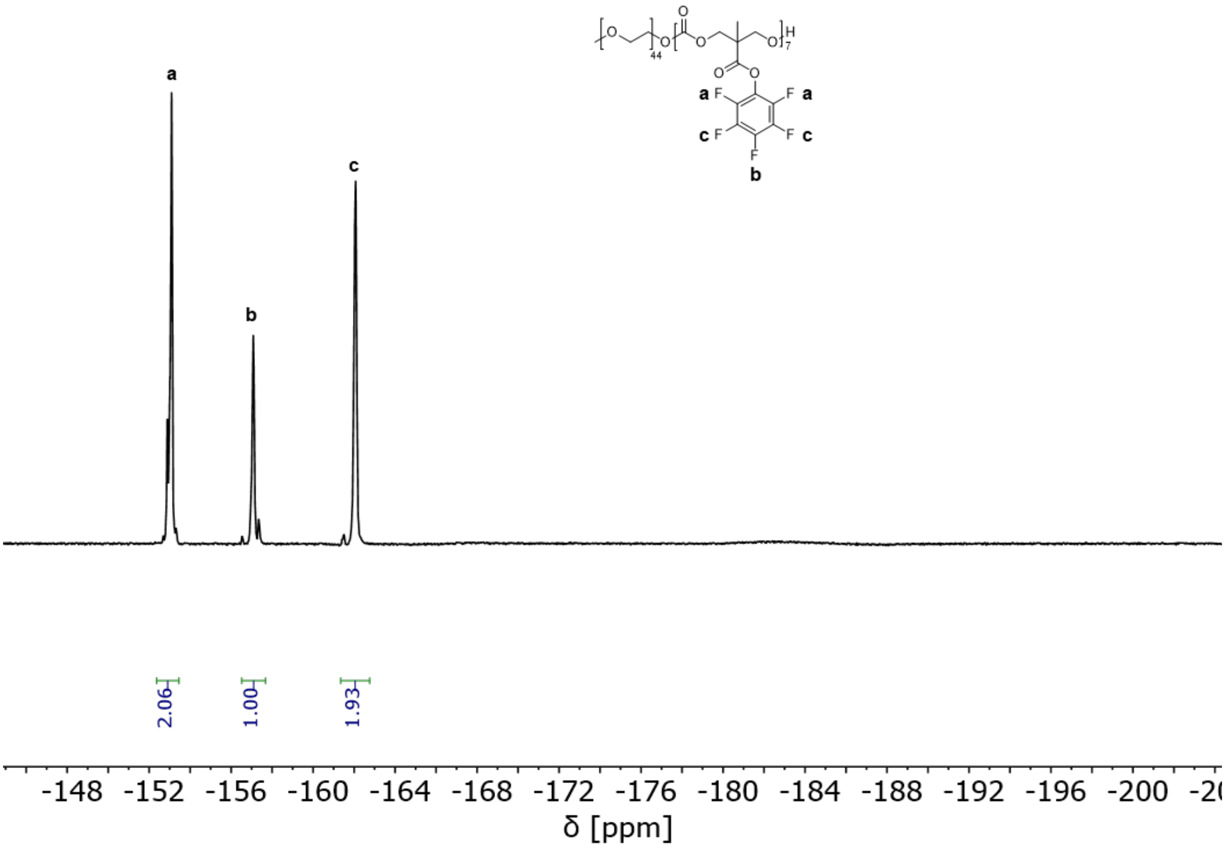

Figure S 19 ^{19}F NMR spectrum of mPEG$_{44}$-*b*-poly(MTC-PFP)$_7$ in $CDCl_3$.

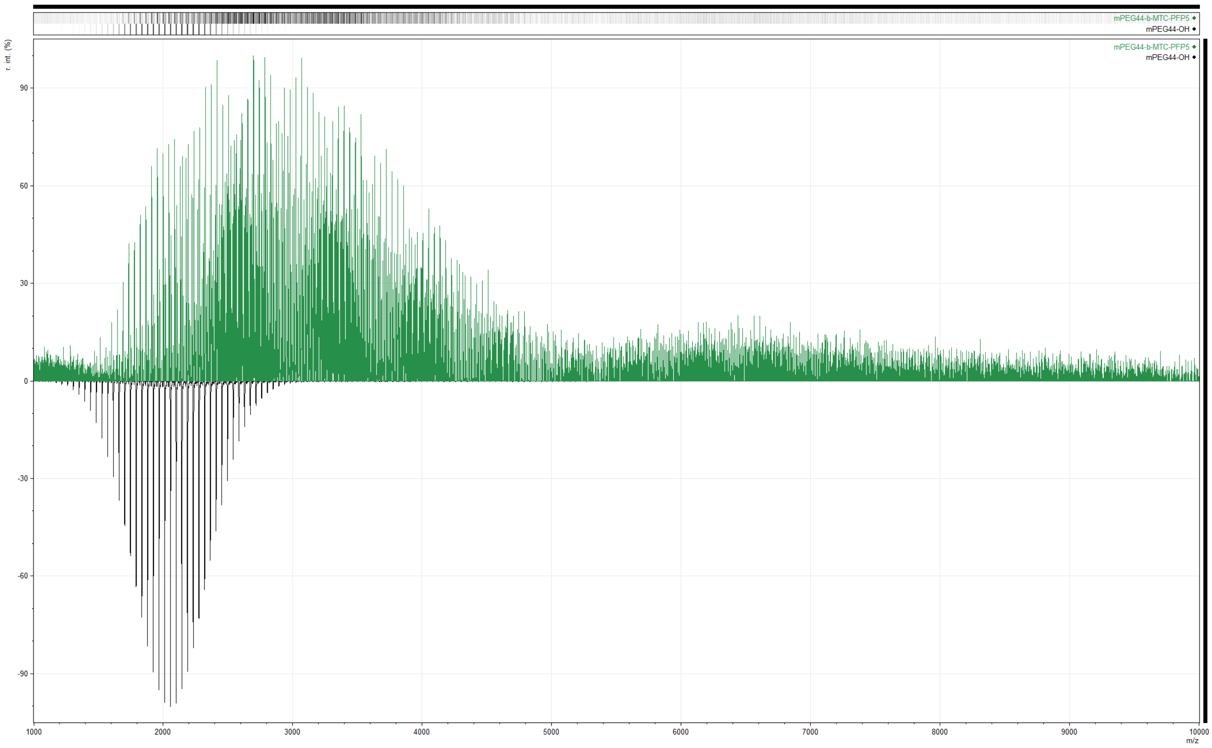

Figure S 20 MALDI-ToF spectrum of mPEG$_{44}$-*b*-poly(MTC-PFP)$_5$ (top, green spectrum) and mPEG$_{44}$ (bottom, black spectrum). MALDI-ToF shows the successful polymerization of the polycarbonate block onto mPEG$_{44}$ and a narrowly distributed spectrum.

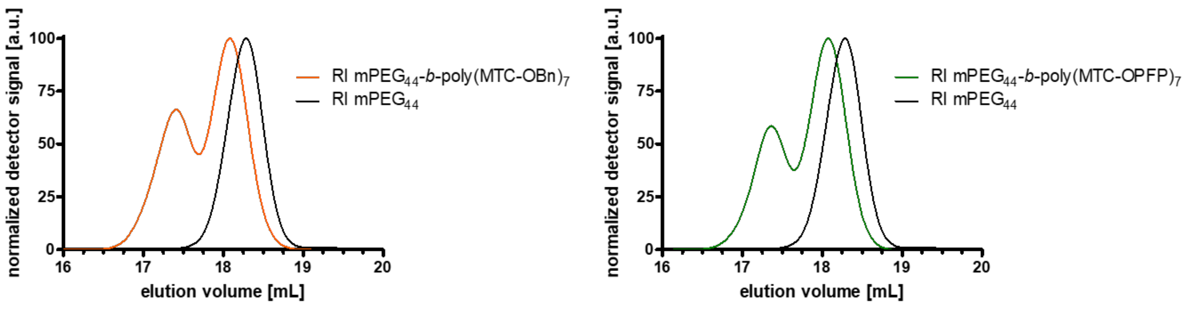

Figure S 21 HFIP SEC elugrams of mPEG$_{44}$-*b*-poly(MTC-OBn)$_7$ and mPEG$_{44}$-*b*-poly(MTC-PFP)$_7$. Both elugrams show a clear shift from the mPEG$_{44}$ macroinitiator towards shorter elution times (higher mass) indicating the successful polymerization of the polycarbonate block. However, a higher molecular species possible arising from transesterification side reaction was also clearly detectable, owning to the required reaction temperatures of +25 °C. Lower temperatures that suppress these reactions are not applicable due to the low solubility of mPEG$_{44}$ in THF at those temperatures.

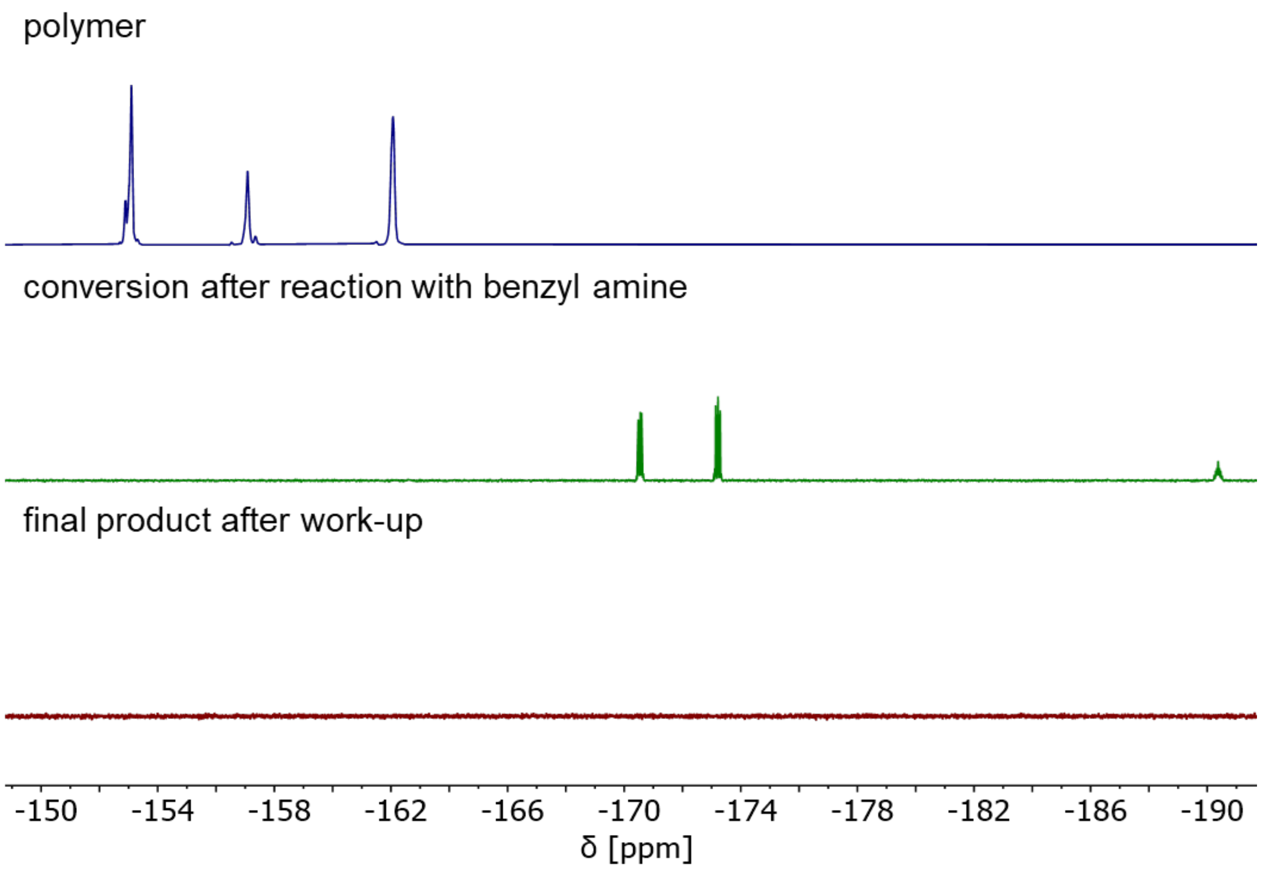

Figure S 22 ^{19}F NMR spectra of post-polymerization modification of mPEG$_{44}$-*b*-poly(MTC-PFP)$_5$ before modification (top) showing polymer bound pentafluorophenyl esters, after conversion with benzylamine (middle) showing released pentafluorophenol and the final product mPEG$_{44}$-*b*-poly(MTC-NHBn)$_5$ (bottom) after work-up by dialysis fully removing the pentafluorophenyl byproduct.

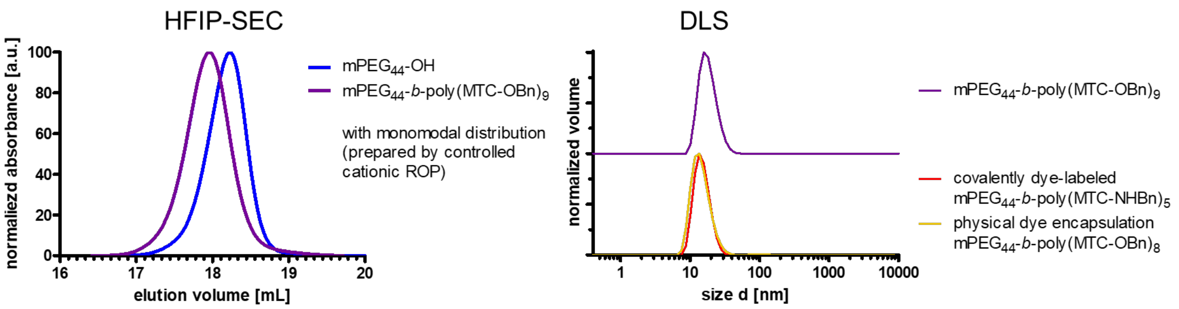

Figure S 23 Block copolymers of MTC-OBn and mPEG44 can also be obtained by DBU-catalyzed ROP and the resulting block copolymers can as well get co-assembled into well-defined block copolymer micelles.[3] The HFIP SEC of $mPEG_{44}$ and $mPEG_{44}$-*b*-poly(MTC-OBn)$_9$ (left) shows successful polymerization of the polycarbonate block and the absence of any higher molecular weight species derived by transesterification reaction (left – compare Figure S21). However, after block copolymer self-assembly similar sizes for the block copolymer micelles can be obtained (right): For $mPEG_{44}$-*b*-poly(MTC-OBn)$_9$ a volume mean of 18.1 nm and a PDI = 0.07 is obtained, while for for $mPEG_{44}$-*b*-poly(MTC-OBn)$_7$ a volume mean of 15.1 nm and for $mPEG_{44}$-*b*-poly(MTC-NHBn)$_5$ a volume 15.3 nm and a PDI = 0.48 are obtained (compare Figure 4). Thus, the obtained transesterification reactions do not significantly affect the micellar self-assembly.

References

[1] S. Saba, A. M. Brescia, M. K. Kaloustian, *Tetrahedron Lett.* **1991**, *32*, 5031.

[2] S. Naumann, A. W. Thomas, A. P. Dove, *ACS Macro Lett.* **2016**, *5*, 134.

[3] C. Czysch, C. Medina-Montano, N. K. Dal, T. Dinh, Y. Fröder, P. Winterwerber, K. Maxeiner, H. Räder, D. Schuppan, H. Schild, M. Bros, B. Biersack, F. Feranoli, S. Grabbe, L. Nuhn, *Macromol. Rapid Commun.* **2022**, 2200095.

[4] R. C. Pratt, F. Nederberg, R. M. Waymouth, J. L. Hedrick, *Chem. Commun.* **2008**, *2*, 114.

[5] T. F. Al-Azemi, K. S. Bisht, *Macromolecules* **1999**, *32*, 6536.

[6] D. P. Sanders, K. Fukushima, D. J. Coady, A. Nelson, M. Fujiwara, M. Yasumoto, J. L. Hedrick, *J. Am. Chem. Soc.* **2010**, *132*, 14724.
